# Activated mTOR Signaling in the RPE Drives EMT, Autophagy, and Metabolic Disruption, Resulting in AMD‐Like Pathology in Mice

**DOI:** 10.1111/acel.70018

**Published:** 2025-02-17

**Authors:** Olivia Chowdhury, Sridhar Bammidi, Pooja Gautam, Vishnu Suresh Babu, Haitao Liu, Peng Shang, Ying Xin, Emma Mahally, Mihir Nemani, Victoria Koontz, Kira Lathrop, Katarzyna M. Kedziora, Jonathan Franks, Ming Sun, Joshua W. Smith, Lauren R. DeVine, Robert N. Cole, Nadezda Stepicheva, Anastasia Strizhakova, Sreya Chattopadhyay, Stacey Hose, Jacob Samuel Zigler, José‐Alain Sahel, Jiang Qian, Prasun Guha, James T. Handa, Sayan Ghosh, Debasish Sinha

**Affiliations:** ^1^ Department of Ophthalmology University of Pittsburgh School of Medicine Pittsburgh Pennsylvania USA; ^2^ Department of Ophthalmology The Wilmer Eye Institute, the Johns Hopkins School of Medicine Baltimore Maryland USA; ^3^ Department of Cell Biology and Center for Biologic Imaging University of Pittsburgh School of Medicine Pittsburgh Pennsylvania USA; ^4^ Johns Hopkins Mass Spectrometry and Proteomics Facility The Johns Hopkins School of Medicine Baltimore Maryland USA; ^5^ Department of Physiology University of Calcutta Kolkata West Bengal India; ^6^ Institut De La Vision INSERM, CNRS, Sorbonne Université Paris France; ^7^ Nevada Institute of Personalized Medicine University of Nevada Las Vegas Nevada USA

**Keywords:** epithelial–mesenchymal transition, metabolic/mitochondrial changes, mLST8, mTOR complex 1, mTOR complex 2, RPE

## Abstract

The mechanistic target of rapamycin (mTOR) complexes 1 and 2 (mTORC1/2) are crucial for various physiological functions. Although the role of mTORC1 in retinal pigmented epithelium (RPE) homeostasis and age–related macular degeneration (AMD) pathogenesis is established, the function of mTORC2 remains unclear. We investigated both complexes in RPE health and disease. Therefore, in this study, we have attempted to demonstrate that the specific overexpression of mammalian lethal with Sec13 protein 8 (mLST8) in the mouse RPE activates both mTORC1 and mTORC2, inducing epithelial–mesenchymal transition (EMT)‐like changes and subretinal/RPE deposits resembling early AMD‐like pathogenesis. Aging in these mice leads to RPE degeneration, causing retinal damage, impaired debris clearance, and metabolic and mitochondrial dysfunction. Inhibition of mTOR with TORIN1 in vitro or βA3/A1‐crystallin in vivo normalized mTORC1/2 activity and restored function, revealing a novel role for the mTOR complexes in regulating RPE function, impacting retinal health and disease.

## Graphical abstract

Our study reveals that overactivation of both mTOR complexes through mLST8 in RPE cells triggers AMD‐like pathology and cellular dysfunction. This finding highlights the critical role of balanced mTOR signaling in retinal health and suggests that targeting both complexes could be therapeutic for AMD treatment.

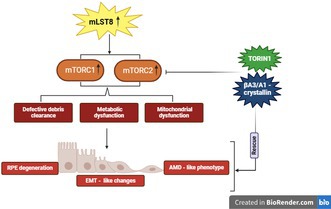

## 1 Introduction

The retinal pigmented epithelium (RPE) is a monolayer of highly polarized, postmitotic cells located at the back of the eye, between the photoreceptors and choriocapillaris (Shang et al. 2018; Lakkaraju et al. 2020). The RPE plays a critical role in maintaining retinal homeostasis through its essential metabolic interactions with the overlying photoreceptors (Strauss 2005). This relationship involves multiple interdependent processes essential for photoreceptor health and optimal retinal function, highlighting the RPE's fundamental importance in vision (Fields et al. 2020). One of the master kinases, mechanistic target of rapamycin (mTOR) (Saxton and Sabatini 2017), is now believed to be an important regulator of RPE health and disease (Go et al. 2020). mTOR exists as complex 1 and complex 2, and can regulate distinct and diverse signaling pathways and cellular function (Saxton and Sabatini 2017). The activation of these complexes is interdependent, allowing for the regulation of key cellular homeostasis processes. Research highlights the essential role of mTORC1 in retinal pigment epithelium (RPE) cells. Zebrafish models demonstrate that mTOR activation drives RPE regeneration by recruiting macrophages and microglia (Lu et al. 2022). In mice, mTOR activation due to mitochondrial dysfunction leads to RPE dedifferentiation and photoreceptor degeneration, effects mitigated by rapamycin (Zhao et al. 2011). Additionally, in diabetic retinopathy, mTORC1 contributes to retinal damage, suggesting that targeting mTOR could be a therapeutic approach for retinal diseases like AMD (Liu et al. 2020). However, the combined function of both mTOR complexes in maintaining RPE health has not been previously investigated.

To investigate the effects of mTOR complex activation in RPE cells, we created a genetically engineered mouse model with RPE‐specific knock‐in (KI) of mammalian lethal with SEC13 protein 8 (mLST8), a key regulator of mTORC1 and mTORC2. mLST8 binds to mTOR's kinase domain, stabilizing it and enhancing mTORC1's kinase activity (Guertin et al. 2006). Moreover, mLST8 helps facilitate substrate binding in mTORC1 and is important for the full activation of downstream signaling pathways, including S6K and 4E‐BP1 phosphorylation (Laplante and Sabatini 2012), which are crucial for cell growth and proliferation. In mTORC1, mLST8 is essential for cell growth and proliferation, whereas in mTORC2, it provides structural support, though its role in mTORC2 signaling is less clearly understood (Guertin et al. 2006). Although mLST8 knockout or knockdown does not affect mTORC1, it is indispensable for mTORC2 function because it is required for the formation of the Rictor–mTOR complex formation during development (Guertin et al. 2006). mLST8 upregulation activates both mTORC1 and mTORC2 in melanoma cells, indicating its regulatory role in both complexes. Similarly, increased mTORC1 and mTORC2 activity in RPE cells has been linked to autophagy abnormalities (Valapala et al. 2014) and induction of an autosomal‐dominant form of Stargardt–like macular dystrophy (Sethna et al. 2021).

We show that mTOR activation in RPE cells disrupts key functions, driving glycolysis and promoting epithelial–mesenchymal transition (EMT), a hallmark of AMD. mTOR regulates EMT through pathways involving reactive oxygen species (ROS), inflammation, and growth factor signaling (Liu et al. 2022; Ghosh et al. 2019). In *mLST8* KI mice, this transition leads to decreased cell–cell adhesion, increased migratory capacity, and subretinal fibrosis formation as they age, disrupting normal retinal architecture and function (Kakumoto et al. 2015; Shang et al. 2021). These changes, coupled with abnormal lysosome‐mediated clearance of cellular debris, result in drusen formation, a characteristic feature of dry AMD. Inhibiting mTOR in the RPE with either a chemical inhibitor (TORIN1) or a biological regulator (βA3/A1‐crystallin) rescued the observed molecular and phenotypic changes, suggesting that targeting the mTOR complex represents a potential therapeutic strategy for dry AMD patients.

## 2 Results

### 2.1 *mLST8* Overexpression in the RPE Leads to Increased Downstream mTORC1 and mTORC2 Activit*y*

**FIGURE 1:**
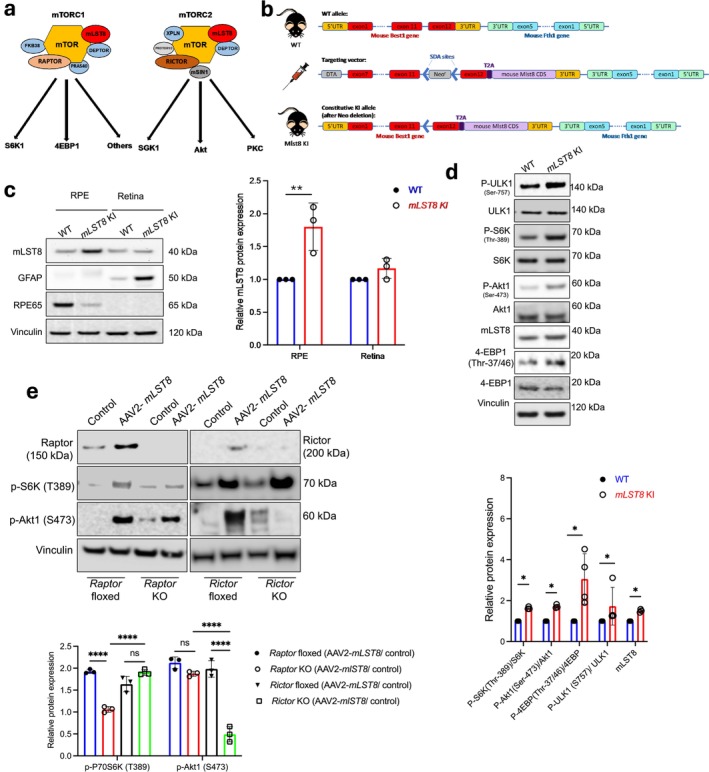
Generation and characterization of *mLST8* KI mice. (a) Cartoon showing that in addition to mTOR, mLST8 is a major component of both mTOR complexes 1 and 2. (b) Schematic showing the strategy for generating *mLST8* KI mice. Briefly, TGA stop codon in the mouse *Best1* gene was replaced with the “T2A‐mouse mLST8 CDS (coding sequence)” cassette. The targeting vector was generated by PCR using BAC clone RP23‐340G9 from the C57BL/6 library as a template. The targeting vector had Neo cassette, which was flanked by SDA (self‐deletion anchor) sites. DTA (diptheria toxin A) was used for negative selection. C57BL/6 embryonic stem cells were used for gene targeting. (c) Western blot analysis indicating that RPE lysates shows overexpression of mLST8 relative to WT. Such changes were not seen in retina lysates from the same mice. Retina RPE lysate preparation was confirmed by evaluating the levels of GFAP and RPE65 in the respective lysates *n* = 3. **p < 0.01. (d) Western blot from RPE lysates of *mLST8* KI RPE cells further revealed an increased ratio of p‐S6K/S6K, p‐4EBP/4EBP, p‐ULK1/ULK1, and p‐Akt1/Akt1 in these cells, compared to controls (WT) *n* = 3. *p < 0.05. (e) Western blot showing that mLST8 overexpression (AAV2‐*mLST8*) in *Raptor* or *Rictor* KO MEF cells activates mTORC2 target (p‐Akt1) and mTORC1 target (p‐P70S6K), respectively, compared to controls *n* = 3. ****p < 0.001, ns = not significant.

mTOR signaling plays a crucial role in RPE physiology (Go et al. 2020) and is the core component of two multi‐subunit complexes mTORC1 and mTORC2 (Saxton and Sabatini 2017). Abnormal mTOR activation in the RPE has been reported in animal models of RPE and retinal degeneration (Go et al. 2020; Valapala et al. 2014). mLST8 is a subunit common to both mTORC1 and mTORC2 that is necessary for activating the mTOR kinase (Figure 1a) (Saxton and Sabatini 2017). The role of mTORC1 and mTORC2 in RPE homeostasis and disease progression is unclear. To investigate the role of both mTOR complexes in RPE cells, we created RPE‐specific *mLST8* KI (to upregulate mLST8 specifically in the RPE) mice using the *Best1* promoter (Figure 1b) for targeted gene overexpression as explained previously (Liu et al. 2022). The RPE‐specific overexpression of mLST8 was confirmed using RPE65 and GFAP as RPE and retina‐specific markers, respectively (Figure 1c). We also observed significant upregulation of GFAP in the *mLST8* KI retina (Figure 1c), a phenotype that we previously showed to be associated with activation of astrocytes and Müller glia (Ghosh et al. 2019). However, we did not find upregulation of mLST8 in the testes (*Best1* is expressed only in the testis apart from the RPE) of *mLST8* KI mice (Figure S1), indicating an RPE‐specific upregulation of mLST8 in these mice.

To assess the contribution of mLST8 upregulation on several mTORC1 and mTORC2 pathway proteins in the RPE cells, we ran a phosphoprotein array to evaluate the levels of phospho‐mTOR (Ser 2448) as well as its immediate downstream targets, phospho‐S6K (Thr 421/Ser 424), and phospho‐Akt (Ser 473). We found increased levels of these proteins as well as phospho‐RPS6 (Ser 235/236) in *mLST8* KI RPE cells, compared to wild‐type (WT) C57BL/6J RPE (Figure S2a,b). We confirmed increased phosphorylation of mTORC1 and mTORC2 downstream substrates, P70S6K, 4EBP, ULK, and Akt1, by western blotting in *mLST8* KI RPE cells as compared with cells from age‐matched WT mice (Figure 1d).

mLST8 may have different functions in the mTOR complexes (Guertin et al. 2006; Kakumoto et al. 2015). Mutations in the *mlst8* gene or its complete ablation disrupts mTORC2 protein assembly, while sparing mTORC1 structure and function (Guertin et al. 2006; Kakumoto et al. 2015). However, mLST8 upregulation has been observed in certain types of cancers, contributing to tumor progression by constitutively activating both mTORC1 and mTORC2 pathways (Kakumoto et al. 2015). In this study, we aimed to ascertain the contribution of mLST8 overexpression on both mTORC1 and mTORC2 activities in RPE cells. Using antibodies to Raptor (mTORC1 complex protein) and Rictor (mTORC2 complex protein), we immuno‐purified Raptor–mTOR (mTORC1) and Rictor–mTOR (mTORC2) from control and mLST8‐overexpressing ARPE19 (undifferentiated) cell lysates and assayed their ability to phosphorylate recombinant P70S6K at Thr 389, an mTORC1 target, and recombinant Akt at Ser 473, an mTORC2 target, respectively. We found increased pAkt1 (Ser 473)/Akt1, p‐ULK1 (Ser 757)/ULK1, p‐4EBP1 (Thr 37/46)/4EBP1, and pP70S6K (Thr 389)/S6K ratios in these undifferentiated ARPE19 cells overexpressing mLST8 compared to control cells (Figure S3). Both mTORC1 and mTORC2 can regulate each other (Saxton and Sabatini 2017), to evaluate if activation of both mTOR complexes upon mLST8 upregulation is dependent on each other or not. We overexpressed an AAV2‐*mlst8* construct in mouse embryonic fibroblasts (MEFs) lacking *Rictor* (*Rictor* KO; mTORC2 protein subunit) or Raptor (*Raptor* KO; mTORC1 protein subunit) with floxed cells as controls (Kakumoto et al. 2015). Our results showed that mLST8 overexpression could induce phosphorylation of P70S6K and 4EBP, but not AKT in *Rictor* KO MEFs and phosphorylation of AKT but not the other mTORC1 target proteins in *Raptor* KO MEFs (Figure 1e). These findings suggest that mLST8 overexpression significantly affects the activity of both mTORC1 and mTORC2, but independently of each other.

### 2.2 RPE‐Specific *mLST8* KI Mice Exhibit Progressive Retinal Pigmentary and Structural Abnormalities Phenocopying Early AMD

**FIGURE 2:**
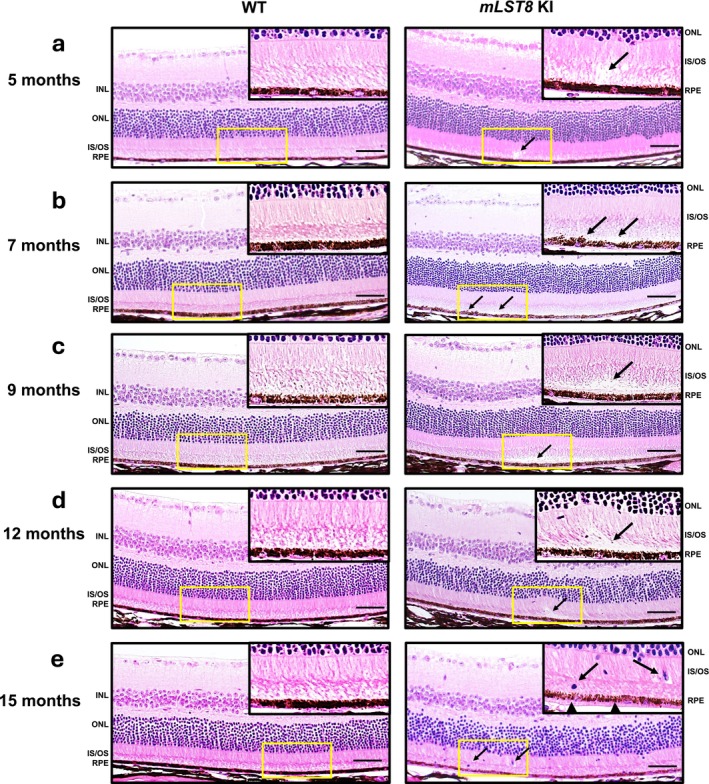
*mLST8* KI mice show retinal structural abnormalities. Hematoxylin–eosin staining on retinal sections revealed disintegration of the POS layer in 5‐month‐old *mLST8* KI retina, but not in age‐matched controls (a, arrows). Consistent abnormalities were also observed in the SRS (disruptions/debris‐like accumulation, cellular infiltrations) and RPE (patchy, depigmented appearance) with increased age in 7, 9, 12 and 15‐month‐old *mLST8* KI retina compared to WT (b, c, d, e) *n* = 6. Scale bar = 50 μm (Zoomed Inset: 20 μm).

Several studies have shown that mTOR activation in the RPE triggers structural and functional abnormalities in both the RPE and neurosensory retina (Valapala et al. 2014; Sethna et al. 2021). As our genetically engineered mice overexpress mLST8 in the RPE, which activates both mTORC1 and mTORC2, it became imperative to examine the RPE for structural and functional variations. Hematoxylin and eosin staining of retinal sections from 5‐month‐old *mLST8* KI mice showed subtle changes in the RPE and the photoreceptor layer, compared to age‐matched WT (Figure 2a). At 7 months of age, *mLST8* KI retinal sections showed disruptions in the RPE and gaps in the subretinal space (SRS) (Figure 2b), whereas at 9 months *mLST8* KI sections showed substantial changes in the SRS (Figure 2c), which persisted at 12 months of age as well (Figure 2d); these changes were not seen in age‐matched WT. At 15 months, there was infiltration of cells (possibly immune cells) in the photoreceptor layer, with significant disruption at the SRS (Figure 2e). These findings are meaningful because we and others have demonstrated that such infiltration is critical for inducing retinal inflammation and subsequent degeneration (Ghosh et al. 2019; Kakumoto et al. 2015). We found increased localization of Iba1‐positive cells (arrows) in the SRS, a region now thought of as to be critical in the transition from parainflammation to chronic inflammation observed in retinal degenerative diseases (Yazdankhah et al. 2023) in *mLST8* KI retinal sections from 9‐month‐old *mLST8* KI mice compared to WT (Figure S4). Interestingly, *mLST8* KI sections also showed a noticeably patchy RPE with loss of pigmentation across all age groups, compared to WT, indicating changes in pigmentary processes in the RPE.

### 2.3 *mLST8* Overexpression Specifically in the RPE Leads to EMT‐Like Changes Resulting in Early AMD‐Like Pathology

**FIGURE 3:**
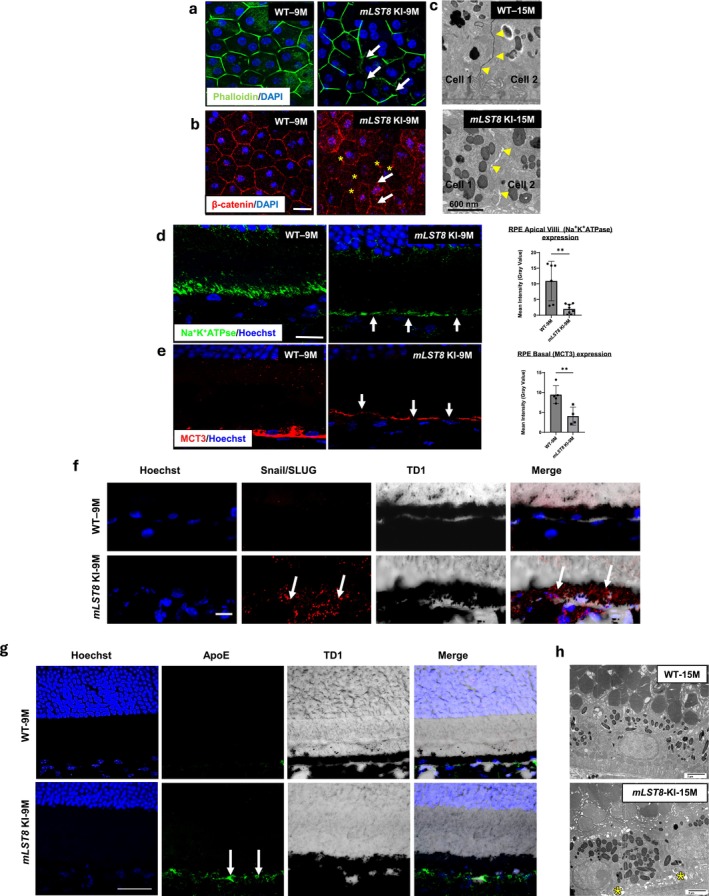
mLST8 KI RPE cells show accumulation of debris and structural abnormalities. (a) Immunostaining of RPE flat mounts with phalloidin‐Alexafluor 488 to stain F‐actin (Green) showed pronounced changes in the honeycomb‐like morphology in 9‐month‐old *mLST8* KI RPE, with noticeable disruption of cell boundaries (arrows) compared to age‐matched controls *n* = 4. Scale bar = 20 μm. (b) β‐catenin staining (red) on RPE flatmounts showed noticeable decrease in the expression in the 9‐month‐old *mLST8* KI RPE cells (arrows), compared to WT, with the increase of cytosolic localization of the protein (asterisks in b) *n* = 4. Scale bar = 20 μm. (c) Transmission electron microscopy reveals a disruption of the tight junctions between adjacent RPE cells in 15‐month‐old *mLST8* KI mice compared to age‐matched WT controls (arrowheads). Scale bar = 600 nm (d) Immunohistochemistry of 9‐month‐old retinal sections showed a disruption of apical polarity characterized by the expression Na^+^K^+^ATPase (green) and basal polarity characterized by MCT3 (red). **p < 0.01. Scale bar = 20 μm. (e) The *mLST8*KI mice show disruption (arrows) as well as significant decrease in the expression of the protein compared to age–matched wild‐type controls. **p < 0.01. Scale bar = 20 μm. (f) Immunohistochemistry of 9‐month‐old *mLST8* KI retinal sections stained positive for EMT markers Snail/SLUG (red) (arrows) compared to WT controls *n* = 4. Scale bar = 20 μm. (g) Immunohistochemistry of 9‐month‐old retinal sections showed basolateral accumulation of early AMD markers, APOE (green) in *mLST8* KI RPE (arrow) but not in age‐matched WT. Scale bar = 20 μm. (h) TEM images show deposits under the RPE cell at the basal lamina in a 15‐month‐old *mLST8* KI (asterisk in d) compared to age–matched WT controls. Scale bar = 600 nm.

Alterations of the retinal and RPE layers prompted us to examine the RPE cell morphology and ultrastructure in the *mLST8* KI mice because RPE monolayer integrity is crucial for its function as a physical barrier between the photoreceptors and the choriocapillaris. In addition, Bruch's membrane also serves as a barrier between retina and choroid (Shang et al. 2018; Lakkaraju et al. 2020). The interepithelial junctional complexes in the RPE maintain cell polarity and prevent intramembranous diffusion between the basolateral and apical membrane domains (Shang et al. 2018, 2021; Lakkaraju et al. 2020). To investigate the morphological integrity of the outer blood–retinal barrier (BRB), we used immunocytochemistry to examine the expression of F‐actin, a tight junction cytoskeletal protein, and β‐catenin on RPE flatmount samples of 9‐month‐old WT and KI mice. WT monolayers displayed a regular cobblestone‐like arrangement highlighted by phalloidin (green) staining of actin that defines the apical boundaries of individual epithelial cells. This normal pattern is distorted in the KI counterpart (Figure 3a). In addition, the occasional, short interruptions in the staining observed in the WT RPE become large and frequent in *mLST8* KI RPE flat mounts, with the phalloidin‐labeled cells becoming visibly distorted (arrows in Figure 3a), suggesting widespread RPE morphologic abnormalities.

In addition to tight junctions, adherens junctions also promote cell adhesion by forming complexes with catenins and actin filaments (Shang et al. 2021). β‐catenin localizes to adherens junctions in normal cells, and its delocalization from the cell membrane to the cytosol and/or nucleus is associated with a cellular stress response (Shang et al. 2021). Overexpression of mLST8 significantly altered the distribution of β‐catenin in KI RPE flat mounts, causing a noticeable shift from the membrane to the cytoplasm (marked by asterisks) compared to the WT (Figure 3b). This suggests that the disruption of cell‐to‐cell connections between RPE cells disrupts the outer BRB (Shang et al. 2021).

To characterize the ultrastructural changes in tight junctions, we performed transmission electron microscopy (TEM) on the eyes of 15‐month‐old KI mice and WT controls. We observed distortions (gaps; yellow arrowheads) in the RPE tight junctions of KI mice, in contrast to the intact tight junctions in WT controls (Figure 3c).

One of the salient features of the RPE cell is its polarity. Polarity is crucial for RPE cells as it enables proper orientation and function, ensuring efficient nutrient transport, waste removal, and maintenance of the BRB essential for retinal health (Lakkaraju et al. 2020). Given the previously observed histological changes, we performed immunohistochemistry studies on 9‐month‐old WT and *mLST8* KI retinal sections to assess alterations in polarity, examining both the apical and basal sides. Na^+^K^+^ATPase maintains the electrochemical gradient by pumping sodium out and potassium into the cell, which is essential for the correct localization of membrane proteins and tight junction integrity (Shang et al. 2021). Monocarboxylate transporter 3 (MCT3), responsible for transporting metabolic byproducts like lactate from the basal side to the choroidal circulation, supports basal polarity and metabolic balance (Shang et al. 2021). Together, these proteins ensure the proper distribution of cellular components and maintain the RPE's structural and functional integrity. Our results showed diminished and mislocalized RPE expression of Na^+^/K^+^‐ATPase (apical marker of RPE) (Figure 3d) and MCT3 (basolateral marker of RPE) (Figure 3e) in the KI mice when compared to WT controls. We observed a marked reduction in the length of apical microvilli in the RPE (stained with phalloidin) and degeneration of the photoreceptor layer, evidenced by a significant decrease in rhodopsin expression (Figure S5). Our histological analysis reveals a transition in the shape and integrity of RPE cells, characterized by a loss of tight junction proteins and subsequent disruption of cell–cell adhesion. By 9 months of age, there is also a noticeable accumulation of extracellular matrix components and fibrotic tissue in the SRS, suggesting progression toward a fibrotic, scar‐like state (Figure 2c). This prompted an investigation into potential EMT in our KI RPE cells. Immunohistochemical staining of 9‐month‐old cryosections for EMT markers Snail and SLUG showed positive staining in the KI group compared to WT controls (Figure 3f). These findings confirm an EMT‐like transition in KI RPE cells, reflecting pathology akin to dry AMD (Ghosh et al. 2018). Furthermore, to ascertain AMD‐like changes in the *mLST8* KI retina, immunohistochemistry on 9‐month‐old WT and *mLST8* KI mouse retinal sections was performed to assess APOE expression, which is prominently observed in human AMD donors as well as mouse models, and is thought to be a critical marker for drusen or drusen‐like deposits in early/dry AMD. We found noticeable staining in the *mLST8* KI RPE, suggesting early AMD‐like changes in these mice (Figure 3g). To confirm that these deposits were contributing to the basal laminar deposits (BLDs) under the RPE, we performed a TEM–based ultrastructural examination and found deposits in the basal laminar area in the KI mice (asterisks in Figure 3h). These results suggest that mLST8 upregulation in RPE triggers an AMD‐like phenotype in mice.

### 2.4 Melanosome Abnormalities in the RPE Are Linked With Lipofuscin Accumulation and Mitochondrial Dysfunction in *mLST8* KI Mice

A key phenotypic change in diseased RPE is with lipofuscin (LF) accumulation, which has been linked to an increase in melanosomes and, in particular, melanin accumulation (Boulton 2014). LF is a complex aggregate of pigmented material resulting from incomplete lysosomal degradation of macromolecules or organelles (Boulton 2014). Increased accumulation of LF granules has been associated with RPE cell death and onset of retinal degenerative diseases, such as AMD and Stargardt's disease (Shang et al. 2017). We confirmed LF accumulation by autofluorescence estimation on RPE flatmounts, which was significantly increased in *mLST8* KI RPE flatmounts (Figure S6a). Using TEM, we found increased accumulation of LF granules in KI samples relative to WT (Figure S6b). In *mLST8* KI RPE cells, melanosomes were redistributed basally (Figure S7a), contrasting with their typical homogenous distribution more towards apical side in WT cells, a defect also observed in Rab27a and myosin‐VIIa mutant (Futter et al. 2004) proteins crucial for melanosome movement via the actin cytoskeleton. The KI RPE displayed melanosome accumulation and abnormal pigmentation (Figure S7b), indicative of lysosomal degradation defects, further supported by reduced co‐localization of the melanosome marker TPC2 and the lysosomal marker LAMP1 in mLST8‐overexpressing cells (Figure S8). Furthermore, RNA sequencing and qPCR confirmed downregulation of melanosome‐related genes such as *Rab27*, *Tyrp1*, and *Mitf* (Figure S9a,b). Additionally, KI RPE exhibited significantly reduced melanin content, linking mLST8 to impaired melanosome function (Figure S9c). Proteomic analysis revealed decreased levels of key melanosome function proteins, including Tyrp1, Mlana, and Bloc1s3 (Figure S10 and Table 1), which was further confirmed by western blot (Figure S11a), whereas mechanistically, mLST8 upregulation caused hyperactivation of mTORC1 and mTORC2, shown by increased phosphorylation of their downstream targets S6K and AKT1 (Figure S11b). Treatment with the mTOR inhibitor Torin1 rescued melanosome marker levels and reduced mTOR signaling, indicating that mLST8 modulates melanosome function via mTORC1/2 activation (Figure S11b). These findings highlight mLST8's essential role in RPE homeostasis. These results demonstrate the abnormal accumulation and altered appearance of melanosomes along with LF accumulation, which could be involved in triggering the retinal degeneration in the *mLST8* KI.

**TABLE 1:** Fold change (*mLST8* KI vs. WT) of melanosome‐related proteins in RPE cell lysates.

Protein accession	Protein	Fold change (mLST8 KI vs. WT)	*p*	Protein sequence coverage
Q2TA50	Mlana	0.3908	0.00304	13%
Q5U5M8	Bloc1s3	0.4579	0.001986	9%
Q9EQJ0	Tpcn1	0.5499	0.114229	1%
A0A1Y7VIZ0	Rab32	0.6188	0.316456	11%
O55102/D3YVM8	Bloc1s1/Bloc1s1	0.6227	0.009389	14%
A0A0R4J075	Mlph	0.7268	0.033828	17%
Q9CWG9/A0A494BAG7	Bloc1s2/Bloc1s2	0.7956	0.676836	19%
P07147	Tyrp1	0.8611	0.027075	16%
Q9ERI2	Rab27a	0.8722	0.562366	24%
Q8VED2	Bloc1s4	0.8951	0.576814	6%
P35279‐2	Rab6a	0.8996	0.566422	22%
P29812	Dct	0.9107	0.038021	12%
Q9CZE3	Rab32	0.951	0.909991	10%
P63011	Rab3a	0.962	0.917582	12%
P61294	Rab6b	0.9806	0.921887	11%
Q9CZB2	Pmel	0.9871	0.972261	4%
Q8QZZ8/A0A140LHK2	Rab38/Rab38	1.097	0.838463	5%
P70280	Vamp7	1.1088	0.77922	5%
P11344	Tyr	1.1614	0.008707	9%
Q62052	Oca2	1.534	0.171486	1%

*Note:* Assessment of the changes in melanosome‐related proteins from mouse RPE lysates. Table showing protein accession number, log_2_ fold change (*mLST8* KI vs. WT), *p*‐values, and % protein sequence coverage for proteins known to be involved in melanosome function and development in cells. *n* = 3.

**FIGURE 4:**
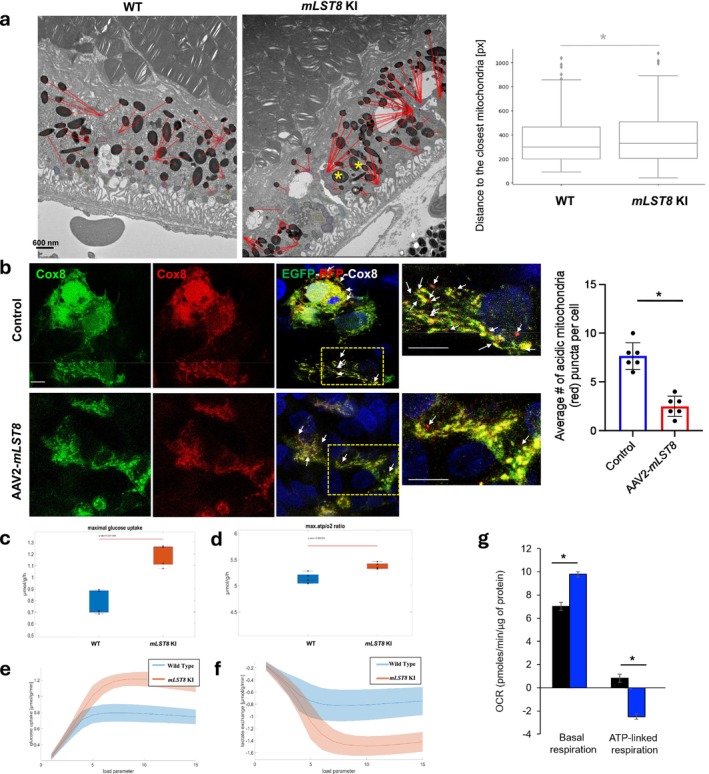
Abnormal mitochondrial function in the RPE cells from mLST8 KI mice. (a) TEM images showing noticeably fewer mitochondria near melanosomes in *mLST8* KI RPE cells compared to WT (asterisks in a). *n* = 3. *p < 0.05. Scale bar = 600 nm. (b) Mitophagy flux was estimated using EGFP‐mCherry‐Cox8 construct in control (uninfected) or AAV2‐*mLST8* construct–infected undifferentiated ARPE19 cells, which showed a reduced number of acidic mitochondria in the mLST8‐overexpressing cells relative to controls, indicating diminished mitophagy in these cells. *n* = 4. Scale bar = 20 μm. *p < 0.05. Metabolomics analysis revealed an increase in (c, e) glucose uptake but decline in (d) ATP production rate and (f) lactate exchange in *mLST8* KI RPE compared to WT, suggesting an abnormal glucose metabolism in these cells. *n* = 5. (g) Seahorse analysis revealed increase in basal respiration but decline in ATP‐linked respiration in cultured adult *mLST8* KI RPE cells compared to WT, indicating abnormal mitochondrial function. *n* = 5. *p < 0.05.

Melanosomes, lysosome‐related organelles from endosomal precursors, mature through stages I–IV with increasing melanin deposition and transport to the cell periphery, requiring high energy. (Boulton 2014; Daniele et al. 2014). As a result, the mitochondria network positions itself adjacent to melanosomes to efficiently supply ATP (Daniele et al. 2014). Mitochondria–melanosome interactions are critical during active melanosome formation and are reduced during melanosome abnormalities (Daniele et al. 2014). Proper melanosome function is essential for RPE health, as they protect against free radicals from high metabolism, phagocytosis, and photooxidative stress, ensuring healthy vision. (Daniele et al. 2014; Sinha et al. 2016). We speculated that alterations in melanosome function in *mLST8* KI RPE would also impact melanosome–mitochondria interactions in these cells. Therefore, we quantified the number of mitochondria in close proximity to melanosomes by quantitatively analyzing TEM images using an artificial intelligence (AI)–based tool and found that the distance to the closest mitochondrion is greater for melanosomes in *mLST8* KI RPE cells than those in WT RPE (Figure 4a). mTOR regulates mitochondrial turnover and mitophagy to maintain cellular health, which may also affect melanosome maturation, highlighting its role in cellular quality control and pigment development in RPE cells (Daniele et al. 2014). To assess mitophagy in mLST8–overexpressing RPE cells, immunofluorescence studies using an EGFP‐mCherry‐Cox8 construct (Rojansky et al. 2016) in undifferentiated ARPE19 cells infected with an AAV2‐*mLST8* construct showed reduced mitophagy, marked by fewer acidic mitochondria (red puncta; arrows in Figure 4b). Metabolomics revealed disrupted glucose metabolism in *mLST8* KI RPE cells, with increased glucose absorption, lower ATP production, and decreased lactate exchange (Figure 4c–f). Mitochondrial function was confirmed by measuring oxygen consumption using the Agilent Seahorse XF Analyzer, showing higher basal respiration but reduced ATP‐linked respiration and decrease in lactate exchange in *mLST8* KI cells (Figure 4g), further proving abnormal glucose metabolism and diminished mitochondrial function in the KI RPE cells.

### 2.5 *mLST8* Overexpression in the RPE Attenuates Macroautophagy

**FIGURE 5:**
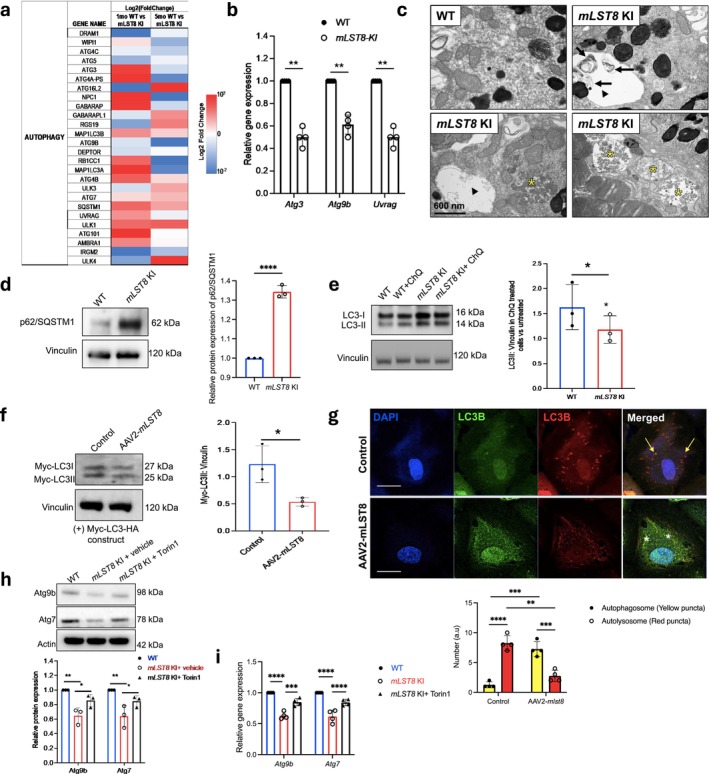
mLST8 overexpression in the RPE triggers alterations in autophagy. (a) Heat map shows differential expression of autophagy‐related genes (Log_2_ fold change; *mLST8* KI vs. WT) from RNAseq analysis of WT and *mLST8* KI RPE cells at 1 and 5 months (*n* = 3). (b) qPCR confirms reduced expression of autophagosome formation genes (*Atg3, Atg9b, Uvrag*) in 5‐month‐old *mLST8* KI RPE cells compared to WT (*n* = 4; **p < 0.01). (c) TEM images reveal increased autophagosomes (double membrane structures, arrows) and debris (asterisks) in *mLST8* KI RPE, indicating impaired clearance, absent in WT (scale bar = 600 nm; *n* = 3). (d) Western blot shows higher p62/SQSTM1 levels in *mLST8* KI RPE (*n* = 3; ****p < 0.0001). (e) Western blot of LC3‐I and LC3‐II in RPE explants with/without chloroquine (ChQ) indicates reduced autophagy flux in mLST8 KI (*n* = 3; *p < 0.05). (f) Undifferentiated ARPE19 cells overexpressing mLST8 show decreased LC3 lipidation compared to controls (*n* = 3; *p < 0.05). (g) AAV2‐mLST8–infected ARPE19 cells (undifferentiated) have increased autophagosomes (yellow puncta, asterisks) and fewer autolysosomes (red puncta, arrows), indicating reduced autophagy flux (*n* = 4; **p < 0.01, scale bar = 10 μm). (h, i) Torin1 treatment (50 nM, 20 h) in *mLST8* KI RPE explants restores autophagy protein and gene levels (Atg7, Atg9b) compared to untreated controls, suggesting upregulation of autophagy mediators *n* = 3. *p < 0.05, **p < 0.01 (for h) and *n* = 4. ***p < 0.001, ****p < 0.0001 (for i).

Our data suggest defective melanosome distribution, clearance, and metabolism in *mLST8* KI RPE, possibly due to reduced clearance from deregulated macro‐autophagy, as seen in a mouse model with abnormal mTORC1 activation in RPE cells. (Valapala et al. 2014). In fact, several studies have implicated autophagy at different stages of melanosome function, metabolism, and particularly AMD pathogenesis (Valapala et al. 2014). Therefore, given that mTOR regulates autophagy (Saxton and Sabatini 2017), we sought to assess the status of autophagy in *mLST8* KI RPE cells. We first performed whole transcriptome sequencing of WT and KI RPE and generated a heat map comparing the expression of autophagy‐related genes between the two groups (Figure 5a). Interestingly, many of the key genes responsible for autophagosome initiation and maturation, such as *Atg3, Atg5, Atg9b, Uvrag, Rb1cc1, Map1lc3a*, and *Gabarap*, were downregulated in the KI RPE, indicating a deregulation of autophagosome formation. To validate these results, we next performed qPCR of a few key autophagosome regulating genes, such as *Atg3, Atg9b*, and *Uvrag*, and observed similar diminished expression (Figure 5b). Furthermore, ultrastructural analysis of the RPE revealed that autophagosomes accumulate in the *mLST8* KI RPE (arrows in Figure 5c) relative to WT cells (Figure 5c). Further, we observed large vacuoles (arrow heads in Figure 5c) and undigested debris in the SRS (below the POS; characterized as an early change in AMD) (yellow asterisks) in *mLST8* KI, but not in WT sections (Figure 5c). Moreover, we also found increased expression of p62/SQSTM1 (Shang et al. 2017) (Figure 5d), a classical receptor for autophagy that is known to play a key role in the process of clearing cellular waste from the cytoplasm. These results imply that the mTOR complexes regulate autophagy in RPE cells.

The degradation of photoreceptor outer segments via phagocytosis/autophagy is crucial for photoreceptor health. LC3, a marker of autophagy flux, undergoes lipidation to form LC3‐II, targeting autophagic membranes and marking autophagosomes (Sinha et al. 2016; Kakumoto et al. 2015; Valapala et al. 2014; Gupta et al. 2022). Given the altered expression of autophagy genes, we next measured the autophagy flux in the RPE of our KI mouse model. To do so, WT and *mLST8* KI RPE explants were cultured with or without chloroquine (ChQ), a lysosomotropic agent, for 6 h followed by western blotting (Gupta et al. 2022). We found significantly decreased autophagy flux (LC3‐II/vinculin ratio in ChQ treated vs. untreated) in the *mLST8* KI RPE explants relative to WT explants (Figure 5e). Further, to determine if the decreased flux could be attributed to impaired LC3 processing/lipidation due to elevated *mLST8*, human ARPE19 cells (undifferentiated) overexpressing mLST8 were transfected with a Myc‐LC3‐HA construct (Kabeya et al. 2000), which identifies autophagosome formation. We found a reduced Myc‐LC3‐II/vinculin ratio in lysates from mLST8‐overexpressing RPE compared to controls (Figure 5f), indicating that mLST8 overexpression impairs LC3 processing, thereby reducing autophagy flux (Gupta et al. 2022; Kabeya et al. 2000). To provide additional evidence that mLST8 alters autophagy flux, human undifferentiated ARPE19 cells were infected with the mLST8‐AAV2 construct followed by overnight infection with adenovirus‐GFP‐RFP‐LC3B, a pH sensitive tandem reporter construct, to label autophagosomes (yellow) and autolysosomes (red) (Valapala et al. 2014; Gupta et al. 2022). In this assay, RFP fluorescence is stable in the acidic endolysosome compartments, whereas GFP fluorescence is rapidly quenched (Valapala et al. 2014; Gupta et al. 2022). The number of red puncta (autolysosomes) was significantly decreased in AAV2‐*mLST8–*infected cells when compared with controls (Figure 5g), whereas the number of autophagosomes (yellow puncta) was significantly increased in mLST8‐overexpressing cells (Figure 5g), which is consistent with decreased autophagy flux in RPE cells overexpressing mLST8 (Figure 5e). To further ascertain if the deleterious effects of mLST8 upregulation on the autophagy pathway in RPE cells could be mitigated by targeting mTOR through mTORC1 and mTORC2 inactivation, we treated *mLST8* KI RPE explants with Torin1 or vehicle (DMSO) overnight. The Torin1‐treated explants had significantly increased protein and gene levels of the autophagy regulators Atg9b and Atg7 (Figure 5h,i).

Finally, to link these structural and molecular abnormalities with functional derangement in the *mLST8* KI mice, we analyzed the relative expression of key RPE–specific visual cycle proteins (Shang et al. 2017), RPE65 and RDH5, and found them to be significantly decreased in KI RPE compared to the WT controls (Figure S12a). Next, we investigated the effect of these visual cycle proteins on the functional status of the RPE and retina by electroretinography. *mLST8* KI mice had decreased c‐wave amplitudes, which is indicative of decreased RPE function (Figure S12b). In addition, the *mLST8* KI mice had a progressive reduction in scotopic a‐wave (Figure S12c,e) and b‐wave (Figure S12d,f) amplitudes with age when compared to age–matched WT mice, indicating a decline in global retinal function.

### 2.6 Subretinal Injection of AAV2‐m*Cryba1* Restores Autophagy and Melanosome Maturation Proteins in *mLST8* KI Mice

Our group previously reported that lack of βA3/A1‐crystallin in RPE cells activates mTORC1 signaling and inhibits autophagy, whereas overexpression of the crystallin reverses these changes (Valapala et al. 2014; Shang et al. 2017). In addition, loss of βA3/A1‐crystallin specifically in the RPE resulted in noticeable melanosome accumulation (Valapala et al. 2014). We found that despite melanosome accumulation in *Cryba1* cKO RPE cells, protein expression analysis showed no significant change in melanosome markers like PMEL and tyrosinase (Valapala et al. 2014; Gupta et al. 2022). These results indicate that βA3/A1‐crystallin is a biological modulator of mTOR and that overexpression of the protein in RPE cells might be beneficial in counteracting the deleterious effects of mTORC1/2 activation.

**FIGURE 6:**
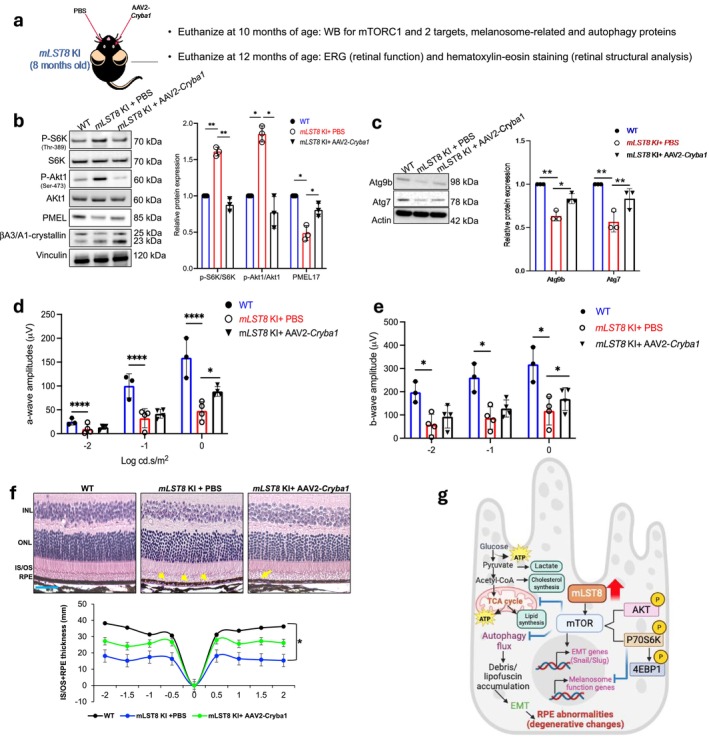
βA3/A1‐crystallin overexpression in the RPE rescues autophagy and melanosome alterations and retinal structure/function in *mLST8* KI mice. (a) Cartoon showing the strategy for subretinal injection of the AAV2‐m*Cryba1* construct into one eye of 8‐month‐old *mLST8* KI mice, with the contralateral eyes receiving PBS vehicle. Animals were euthanized 2 and 4 months after injection. (b, c) Western blot analysis and densitometry showing that *Cryba1* overexpression in the RPE (confirmed by western blot in b) of *mLST8* KI mice could rescue the abnormal levels of both mTORC1 (p‐S6K) and mTORC2 (p‐Akt1) targets (b), and the melanosome marker (PMEL; b), as well as rescued the levels of major regulators of autophagosome formation (Atg9b, Atg7; c) in these animals (b, c), relative to PBS injected eyes (b, c). *n* = 3. *p < 0.05, **p < 0.01. AAV2‐m*Cryba1* treatment (subretinal injection) to *mLST8* KI mice for 4 months rescued retinal function as evident from increase in scotopic (d) a‐ and (e) b‐wave amplitudes after the treatment, compared to PBS‐injected contralateral eyes of the KI mouse. *n* = 4. ****p < 0.0001, *p < 0.05. (f) AAV2‐m*Cryba1* treatment to *mLST8* KI mice for 4 months also rescued early RPE changes (arrows) like the patchy appearance of the monolayer and decline in thickness (spider plot), compared to PBS–treated contralateral eyes of the *mLST8* KI mouse. *n* = 4. Scale bar = 20 μm. *p < 0.05. (g) Cartoon depicting overexpression of mLST8 in RPE cells (*mLST8* KI mice) activated both mTORC1 and mTORC2, disrupting glucose metabolism, mitochondrial function, autophagy, and melanosome function, leading to debris accumulation, EMT activation, and age‐related retinal degeneration resembling AMD. Targeting mTOR with inhibitors or modulators rescued these changes, suggesting a potential therapeutic strategy for retinal diseases by modulating mTOR signaling. Created with BioRender.com.

The benefit of Torin1 on *mLST8* KI RPE (Figures 5h,i and S11b) encouraged us to investigate the effects of Torin1 and βA3/A1‐crystallin treatment in vivo. βA3/A1‐crystallin is known for its protective role in maintaining cellular homeostasis under stress, and recent data suggest its potential to modulate pathways associated with oxidative stress, lysosomal function/autophagy, apoptosis, and inflammation, all of which are critical in the pathogenesis of AMD (Boya et al. 2023). Additionally, Torin1 has been shown to inhibit mTORC1 and also mTORC2 at higher concentrations, a feature not shared by rapamycin, thereby providing broader suppression of mTOR signaling pathways (Thoreen et al. 2020). This broader inhibition could be particularly beneficial in diseases like AMD, where mTORC2 also likely plays a key role in regulating cellular metabolism and survival, potentially influencing disease progression. Unlike rapamycin, which primarily binds to the FKBP12 protein and inhibits mTORC1 activity by allosteric modulation, Torin1 directly competes with ATP binding at the catalytic site of mTORC1, leading to more robust and sustained inhibition of downstream mTOR signaling (Thoreen et al. 2020). This makes Torin1 a potentially more effective therapeutic agent. Although rapamycin has shown antiangiogenic potential in AMD, clinical results have been inconsistent due to factors like limited bioavailability and off‐target effects (Viana et al. 2018; Wu et al. 2022). Additionally, immunosuppressive properties of rapamycin (and rapalogs?) raise safety concerns in elderly patients (Groth et al. 1999). However, reports have also suggested that Torin1, as an mTOR inhibitor, globally attenuates mTOR function and depending on the site of exposure, can lead to severe systemic consequences (Sun 2013). We previously reported that *Cryba1* overexpression in *Cryba1* cKO RPE cells rescued the elevated mTOR activity (Valapala et al. 2014). This result led us to proceed with a single subretinal injection of AAV2*‐mCryba1* to 8‐month‐old (after retinal changes have been established) *mLST8* KI mice (Figure 6a). The mice were euthanized at 10 months of age and autophagy (Atg9b, Atg7), and melanosome (PMEL) markers were analyzed. In addition, a cohort of mice was subjected to evaluation of retinal function and structural changes with or without AAV2‐m*Cryba1* treatment at 12 months of age. We found that overexpression of *Cryba1* rescued the levels of autophagy and melanosome markers in *mLST8* KI RPE lysates (Figure 6b,c). These results were likely due to the ability of *Cryba1* to regulate mTOR signaling in the RPE because we also observed significant reduction of both mTORC1 and mTORC2 target proteins p‐S6K (T389) and pAkt1 (S473) in RPE lysates from *mLST8* KI mice injected with the AAV2‐m*Cryba1* construct compared to PBS‐injected eyes (Figure 6b). Further, retinal function (a‐ and b‐wave amplitudes) and early RPE changes (reduced IS/OS+RPE thickness due to the patchy appearance with intermittent gaps in the monolayer; arrows) were rescued in *mLST8* KI mice 4 months after AAV2‐m*Cryba1* treatment, relative to PBS‐treated contralateral eyes (Figure 6d–f). mLST8 overexpression in RPE cells upregulates mTORC1/2, causing autophagy deregulation, metabolic alterations, and reduced melanosome function, leading to retinal degeneration. Inhibition of mTOR with a chemical inhibitor (Torin1) or biological regulatory protein (βA3/A1‐crystallin) rescued these pathways (Figure 6g).

## 3 Discussion

Our study provides novel insights into the role of mTOR signaling in RPE homeostasis and its potential involvement in dry AMD pathogenesis. By generating a mouse model with RPE‐specific constitutive KI of mLST8, a subunit essential for both mTORC1 and mTORC2, we observed significant retinal and RPE abnormalities leading to retinal degeneration. Previous studies have shown that upregulation of mLST8 induces the activation of both mTORC1 and mTORC2 in multiple cell types (Saxton and Sabatini 2017), simultaneously leading to deficits in melanin biosynthesis, melanosome development, and metabolic abnormalities in cancer and skin pigmentation diseases (Guertin et al. 2006; Kakumoto et al. 2015). In some cancer tissues/cells, mLST8 is upregulated; however, knockdown of mLST8 had no effect on noncancerous epithelial cells (Kakumoto et al. 2015). mLST8 is an essential component of mTORC2 required in mid‐gestational development, as mLST8 KO mice die around e10.5 (Guertin et al. 2006). However, the specific role of these mTOR complexes in regulating RPE function remained poorly understood. Our work bridges this knowledge gap by demonstrating that mLST8‐driven activation of mTORC1 and mTORC2 in the RPE alters cellular physiology.

In our *mLST8* KI mouse model, we observed elevated activities of both mTOR complexes, accompanied by decreased autophagy flux. This abnormal activation of the mTOR complexes, driven by the upregulation of mLST8, disrupted the homeostatic balance within RPE cells, leading to the initiation and progression of EMT, as evidenced by the expression of EMT‐associated transcription factors such as Snail and SLUG. The loss of epithelial characteristics, including the disruption of tight junctions and adherens junctions, resulted in the breakdown of the RPE monolayer. This impairment of the RPE barrier function is a critical event, as it compromises the ability of the RPE to maintain the homeostatic environment of the retina and support the survival and function of photoreceptors. Furthermore, the increased migratory capacity of RPE cells during EMT contributed to the formation of subretinal fibrosis, further disrupting the normal architecture and function of the retina.

Lysosomes play a critical role in regulating melanosome numbers and maintaining cellular homeostasis during aging (Valapala et al. 2014). Previous studies have shown that abnormal lysosomal function leads to accumulation of melanosomes in RPE cells (Valapala et al. 2014). In the *mLST8* KI mice, the accumulation of abnormal or dysfunctional melanosomes in the RPE likely contributed to the induction of LF accumulation and the subsequent degenerative changes observed in the RPE and retina (Boulton 2014). Additionally, our studies revealed mitochondrial and metabolic abnormalities in the *mLST8* KI RPE cells, as evidenced by alterations in ATP formation, glucose metabolism, and mitochondrial function. This is further supported by the rescue of retinal structure and function in our *mLST8* KI mice through βA3/A1‐crystallin overexpression, confirming the importance of the mTOR signaling pathway in these critical molecular changes. Our findings align with and extend previous observations in *Cryba1* cKO mice, where RPE dysfunction occurs with age and is associated with mTORC1 activation in RPE cells.

The novelty of our work lies in elucidating the specific mechanisms by which dysregulated mTOR signaling, driven by mLST8 upregulation, disrupts RPE homeostasis and induces EMT, leading to the development of early AMD‐like pathological features. Unlike previous studies that focused primarily on mTORC1 or general mTOR inhibition, our model allows for the simultaneous examination of both mTORC1 and mTORC2 in the context of RPE function and AMD pathogenesis. This comprehensive approach provides a strong understanding of the complex interplay between mTOR signaling components in retinal health and disease. Our findings not only advance the understanding of molecular pathways involved in AMD pathogenesis but also highlight the potential of targeting the mTOR signaling pathway for therapeutic interventions in retinal degenerative diseases. However, further in‐depth longitudinal studies are needed to fully elucidate the role of both mTOR complexes in the pathogenesis of dry AMD and to translate these findings into clinical applications. This work sets the stage for future investigations into the differential roles of mTORC1 and mTORC2 in RPE function and opens new avenues for developing targeted therapies for AMD and related retinal disorders.

## 4 Materials and Methods

### 4.1 Antibodies

Primary antibodies are as follows: phospho‐p70 S6 kinase (Thr389) (Cell Signaling Technology, 9205), p70 S6 kinase (Cell Signaling Technology, 9202S), phospho Akt1(Ser473) (Cell Signaling Technology, 9018S), Akt1 (Cell Signaling Technology, 2938S), Atg9b (Thermo Fisher Scientific, PA5‐20998), Atg7 (Cell Signaling Technology, 8558S), PMEL17 (Novus Biologicals, NBP1‐69571), p62/SQSTM1 (Novus Biologicals, NBP1‐42821), TYRP1 (Thermo Fisher Scientific, PA5‐107304), Rictor (Bethyl Laboratories, A300‐459A), Raptor (Bethyl Laboratories, A300‐553A), mTOR (Cell Signaling Technology, 2983S), LC3B (Cell Signaling Technology, 2775S), Vinculin (Abcam, ab129002), β‐actin (Cell Signaling Technology, 4970S), GBL/MLST8 (Invitrogen, PA5‐78558), GFAP (Biologicals, NBP1‐05197), RPE65 (Thermo Scientific, PA5‐110315), RDH5 (Thermo Fisher Scientific, PA519319), and βA3/A1‐crystallin (Abcam, ab151722). The secondary anti‐rabbit (7074S) and anti‐mouse (7076S) antibodies were purchased from Cell Signaling Technology.

### 4.2 Generation and Validation of RPE‐Specific *mLST8* KI Mice

RPE–specific *mLST8* KI mice were generated by Cyagen (Santa Clara, CA). Briefly, T2A sequence followed by *MLST8* coding sequence (CDS) was inserted between the last exon and the 3′ untranslated region (3′UTR) of the mouse *Best1* gene. The T2A sequence enables the co‐expression of both *Best1* and *MLST8* from the same *Best1* promoter, which is specific to the RPE. The Neo^r^ cassette flanked with the self‐deletion anchors (SDAs) was inserted in the intron area between exons 11 and 12 of the mouse *Best1* to be deleted in germ cells (Liu et al. 2022). *mLST8* KI was validated by Sanger sequencing. RPE lysates were used to biochemically (western blot) validate RPE‐specific overexpression of mLST8 protein (Figure 1c). All animals including male and female were housed in ventilated microisolator cages under a 12/12 h of light and dark cycle. The temperature and humidity were controlled. All animals studied were conducted in accordance with the Guide for the Care and Use of Animals (National Academy Press) and were approved by the Institutional Animal Care and Use Committee of the University of Pittsburgh under protocol number 20108281.

### 4.3 Electroretinography

WT and *mLST8* KI mice were subjected to dark adaptation for 24 h and were anesthetized (intraperitoneal injection) with 100 μL of a ketamine (50 mg/kg body weight) and xylazine (10 mg/kg body weight) mixture, and then subjected to electroretinography to evaluate retinal function by estimating the scotopic a‐, b‐ and c‐wave responses using the Celeris Diagnosys System (USA) (Liu et al. 2022; Gupta et al. 2022).

### 4.4 Fundus Autofluorescence

Fundus photographs were obtained from anesthetized WT and *mLST8* KI mice using the Micron IV Laser Scanning Ophthalmoscope (Phoenix Research Lab Inc) as previously described (Valapala et al. 2014).

### 4.5 Hematoxylin–Eosin Staining

Eyes from WT and *mLST8* KI mice were fixed in 2.5% glutaraldehyde and then moved to formalin. The tissues underwent alcohol dehydration and were embedded in methyl methacrylate. Sections (1 μm thick) were cut, stained with hematoxylin and eosin, and examined under a light microscope (Ghosh et al. 2019).

### 4.6 Phosphoprotein Array

RPE lysates from WT and *mLST8* KI mice were subjected to a commercially available phosphoprotein array following the manufacturer's protocol (RayBiotech, AAH‐AKT‐1‐2). The levels of all the proteins were normalized to the positive control (POS) and analyzed by ImageJ software.

### 4.7 RPE Explant Preparation

After euthanizing 3‐month‐old WT and *mLST8* KI mice, RPE explants were harvested as previously described (Shang et al. 2018; Gupta et al. 2022). The explants were cultured in complete media, facing up. *mLST8* KI flat mounts were treated with Torin1 (50 nM for 24 h) and infected with adenovirus‐βA3/A1 (Ad‐RFP‐m‐Cryba1[L]; Vector Biolabs, 2001) at a dose of 1 × 10^7^ vg/mL (MOI: 133) for 48 h. The RPE cells were then lysed, and Western blots were performed as previously described (Shang et al. 2018; Gupta et al. 2022).

### 4.8 RPE Flatmount Preparation and Immunofluorescence Studies

Eyes were enucleated and immediately fixed in 4% paraformaldehyde for 1 h at room temperature. Under a dissecting microscope, the anterior segment was removed by a circumferential cut behind the limbus, followed by careful separation of the neural retina from the underlying RPE/choroid complex. To create a flat preparation, four radial incisions were made in the eyecup, allowing the tissue to be flattened into a four leaved “clover leaf” configuration. The RPE was then permeabilized in 0.3% TritonX100 for 1 h and blocked in PBS containing 5% normal goat serum for 1 h at room temperature. Immunostaining was performed using either phalloidin (Thermo Fisher, A12379) to visualize the F‐actin cytoskeleton or β‐catenin antibody (Abcam, ab16051) to examine adherens junctions. The flat mounts were then mounted on a microscope slide with RPE layer up and images were acquired using a Zeiss LSM710 confocal microscope.

### 4.9 Mouse Retinal Cryosectioning

Eyes from freshly euthanized 9‐month‐old WT and *mLST8* KI mice were fixed and cryosectioned (Ghosh et al. 2019; Shang et al. 2021). Slides were incubated overnight at 4°C with a primary antibody cocktail (1:200 dilution) containing rhodopsin (Abcam, ab98887), Iba1 (Wako Chemicals, 019–19741), MCT3 (Alomone Chemicals, AMT‐013), and Na+/K + ‐ATPase (Abcam, ab76020) in a buffer with 1% Tween 20 and 0.5% BSA in PBS. After three washes, a secondary antibody cocktail (1:500 Anti‐Rabbit Alexa Fluor 555) was applied, along with Hoechst or Alexa Fluor 488‐phalloidin (Ghosh et al. 2024; Ghosh et al. 2019; Shang et al. 2021). After additional washes, slides were mounted with the DAKO medium for imaging on an Olympus IX81 confocal microscope. The expression of proteins and the length of phalloidin‐positive microvilli were quantified using ImageJ software (Ghosh et al. 2019; Shang et al. 2021).

### 4.10 ELISA For Melanin Content Estimation

Melanin content was estimated from RPE lysates using a commercially available kit (Biorbyt, orb782359) following the manufacturer's instructions.

### 4.11 TEM and Quantification

Mouse eyes were fixed in cold 2.5% glutaraldehyde and 2% paraformaldehyde in 0.01 M PBS (pH 7.3). The posterior segments, including the sclera, choroid, and retina, were dissected, quartered, and the optic nerve excised. Specimens were rinsed in PBS, post‐fixed in 1% osmium tetroxide with 1% potassium ferricyanide for 1 h, and dehydrated through a graded series of ethanol and propylene oxide before embedding in Poly/Bed 812 resin.

Semi‐thin (300 nm) sections were cut and stained with 0.5% toluidine Blue for light microscopy, whereas ultrathin sections (65 nm) were stained with 2% uranyl acetate and Reynolds lead citrate for analysis using a JEOL 1400 Flash transmission electron microscope. Melanosomes were quantified manually in a blinded manner using ImageJ software (Lee et al. 2022). To assess melanosome–mitochondria proximity, 16 TEM images (10 WT RPE and 6 *mLST8* KI RPE, each from a different mouse) were analyzed. Mitochondria were detected using the MitoNet segmentation network in the Empanada Napari plugin (Conrad and Narayan 2023) and melanosomes with the Cellpose segmentation algorithm (Stringer et al. 2021; Pachitariu and Stringer 2022), calculating distances between organelles based on centroids.

### 4.12 RNAseq Analysis

RPE cells from WT and *mLST8* KI mice at 1 and 5 months of age were subjected to RNAseq and bioinformatic analysis, which was performed as paid service from Novogene, United States.

### 4.13 Subretinal Injection

Eight‐month‐old *mLST8* KI mice were injected subretinally in one eye with AAV2‐hRPE(0.8)‐GFP‐P2A‐m*Cryba1* (Vector Biolabs, 17012305), whereas the contralateral eyes received PBS as explained previously (Gupta et al. 2022). The first group of mice was euthanized 2 months postinjection (at 10 months of age), and their RPE cells were lysed for western blot analysis. The second group was maintained for 4 months postinjection (at 12 months of age) for ERG assessment, followed by euthanasia and H&E staining to evaluate RPE and retinal morphology.

### 4.14 Western Blot

Western blot analysis was performed using previously published methods from our laboratory (Shang et al. 2021). RPE lysates were used and western blots were performed using appropriate primary and secondary anti‐Rabbit (Cell Signaling Technology, 7074S) or anti‐Mouse secondary (Cell Signaling Technology, 7076S) antibodies, respectively, and the protein bands were quantified relative to the loading control (actin or vinculin) using Image J software (National Institutes of Health, USA) (Shang et al. 2021; Gupta et al. 2022).

### 4.15 Culture of iPSC‐Derived RPE Cells and Quantitative Immunostaining to Measure Lysosome–Mediated Melanosome Clearance

iPSC‐derived cells (Fuji Cellular Dynamics, R1101) were grown in differentiating media for 28 days. AAV2‐*mLST8* (106 vg/mL) was used to infect the cells after 7 days. Immunostaining was performed for melanosome marker, TPC2 (Novus Biologicals, NBP1‐86923), and lysosomal membrane protein, LAMP1 (Abcam, ab24170), as explained earlier (Shang et al. 2021; Gupta et al. 2022) and then co‐localization was quantified by using the JaCoP plugin of ImageJ (Gupta et al. 2022).

### 4.16 Autophagy Flux Assessment

Autophagy flux in RPE cells was assessed as previously described (Gupta et al. 2022). Briefly, WT and *mLST8* KI RPE explants were treated with ChQ (50 μM) or left untreated for 6 h. Cells were lysed in 1X RIPA containing 0.1% each of protease and phosphatase inhibitor cocktails and western blot was performed to evaluate the levels of LC3‐I and LC3‐II. Autophagy flux was estimated by calculating the ratio of LC3‐II in ChQ‐treated cells with respect to untreated cells (Gupta et al. 2022).

### 4.17 Evaluation of LC3 Lipidation

ARPE19 cells grown to 80% confluency were transfected with pCI‐neo‐myc‐LC3‐HA (Addgene, 45244) (Kabeya et al. 2000) using the Lipofectamine 3000 transfection kit (Thermo Fisher, L3000015). The medium was changed after 16 h, and to simulate in vivo mLST8 overexpression conditions, cells were either left uninfected or infected with AAV2‐hVMD2‐*hMLST8*‐FLAG (Vector Biolabs, 70210) at a dose of 1 × 10^7^ vg/mL for 48 h. Cells were then lysed and western blot was performed for myc, and LC3 lipidation was estimated in control (uninfected) and AAV2‐hVMD2‐*hMLST8*‐FLAG‐infected cells by evaluating the ratio of Myc‐LC3‐II to loading control (Vinculin) as explained previously (Kabeya et al. 2000).

### 4.18 Estimation of Autophagosome Number in RPE Cells

ARPE19 (undifferentiated) cells were either infected with AAV2‐hVMD2‐*hMLST8*‐FLAG (Vector Biolabs, 70210) at a dose of 1 × 10^7^ vg/mL for 48 h or left uninfected. Then both the control and mLST8 overexpressing cells were infected for 12 h with 10^8^ vg/mL of an adenovirus‐GFP‐RFP‐LC3 construct (Vector Biolabs, 2002) and then fixed with 2% PFA for 30 min at 4°C followed by staining of nuclei with Hoechst (Sigma, B2883) 1 mg/100 mL dH_2_O. Confocal images were acquired at 60× objective using a Zeiss LSM710 microscope. Positive structures for size, shape, and intensity for RFP/GFP, and RFP only puncta were identified, and RFP puncta were quantified as explained previously (Gupta et al. 2022).

### 4.19 Assessment of Mitophagy in RPE Cells

ARPE19 cells (undifferentiated) were infected with AAV2‐hVMD2‐*hMLST8*‐FLAG construct or left untreated. Both the control and mLST8 overexpressing cells were subjected to infection with adenovirus‐Cox8‐EGFP‐mCherry construct (Vector Biolabs, 2001) at a dose of 10^8^ vg/mL for 12 h. The cells were then fixed with 2% PFA for 30 min at 4°C followed by nuclei staining with Hoechst (Sigma, B2883) 1 mg/100 mL dH_2_O. Confocal images were acquired at 60× objective using a Zeiss LSM710 microscope. Red puncta indicating autophagosomes (mitochondria positive) fused to the lysosomes were counted as explained previously (Yazdankhah et al. 2021).

### 4.20 Mass Spectrometry Analysis

Protein extracts (25 μg) were processed using SP3 paramagnetic beads (GE Healthcare) (Hughes et al. 2019). Disulfide bonds were reduced with 50 mM dithiothreitol for 1 h at 60°C, cooled to RT, and alkylated with 100 mM chloroacetamide in the dark for 15 min. SP3 beads (5 μL, 1:1 ratio of GE45152105050250 and GE65152105050250, Millipore Sigma) were added along with 100% ethanol (100 μL). After 5 min of mixing, beads were washed three times with 80% ethanol. Proteins were digested on‐bead with trypsin/lys‐C (Pierce) at 37°C overnight. The supernatant was acidified with 5 μL of 1% formic acid before mass spectrometry.

Peptides (10%, 2.5 μg) were analyzed via reverse‐phase nanoflow chromatography on a Neo Vanquish uHPLC interfaced with an Orbitrap Exploris 480 mass spectrometer (Thermo Fisher Scientific). Peptides were separated over 120 min using a 0%–100% acetonitrile gradient. MS scans were acquired from 350 to 1800 m/z at 120,000 resolution with an AGC of 1e6 and maximum injection time of 25 ms. Data‐independent acquisition (DIA) of MS/MS spectra was done with precursor ions isolated in 24 m/z windows, using HCD activation (30% collision energy), and analyzed at 60,000 resolution with 1e6 AGC and 118 ms injection time.

Raw files were converted to.mzml (Chambers et al. 2012) and analyzed with MSFragger‐DIA via FragPipe (Yu et al. 2023). Spectra were searched using MSFragger (Kong et al. 2017; Teo et al. 2021) against UP589_m_musculus.FASTA with a 10 ppm precursor tolerance and 20 ppm fragment ion tolerance. Variable modifications included deamidation, oxidation, and formylation, with carbamidomethylation as a fixed modification. Peptide–spectrum matches (PSMs) were validated by Percolator (Käll et al. 2007) and proteins inferred by ProteinProphet (Nesvizhskii et al. 2003), with FDR maintained at 1% (da Veiga Leprevost et al. 2020). A spectral library was built with EasyPQP (MacLean et al. 2010).

Search results were imported into Skyline (version 22.2) (Demichev et al. 2022) for protein quantification using fragment ion peak areas normalized to the total ion chromatogram (TIC). Fold changes between *mLST8* KI and WT groups were analyzed with a significance threshold of *α* = 0.05.

### 4.21 mTORC1 And mTORC2 Activity Assay

ARPE19 cells (undifferentiated) were overexpressed with AAV2‐hVMD2‐*hMLST8*‐FLAG construct or left untreated. Following 48 h of the infection with the mLST8 construct, both control and mLST8‐overexpressing cells were starved with serum‐free medium overnight or starved and then refed with a complete medium for 10 min. The cells from all the experimental groups were lysed in the CHAPS lysis buffer, and Raptor and Rictor antibodies were used to immunoprecipitate mTOR bound complexes using protein Ig beads (Thermo Fisher, 88802). mTORC1 and mTORC2 target proteins S6K1 (Sino Biological, 10099‐H09B‐50) and Akt1 (EMD Milipore, 14–279) were given to the immune‐precipitated complexes and incubated for 15 min in 37°C. Western blot was then performed for Raptor and Rictor proteins to establish appropriate pull down of these complexes as well as total and phosphorylated levels of S6K1 and Akt1 in each condition (Guertin et al. 2006).

### 4.22 Measurement of Mitochondrial Function

Primary mouse RPE cells were isolated from posterior eyecups using enzymatic digestion with 2% dispase–0.05% trypsin for 45 min at 37°C. The RPE monolayer was gently separated from the underlying choroid using an angled crescent knife and cultured in 96‐well Seahorse microplates (Agilent) using alpha‐MEM supplemented with 10% FBS, N1 supplements, nonessential amino acids, sodium pyruvate (1 mM), and glutamine (2 mM). After reaching confluence in 5–6 days, cellular metabolism was assessed using the Seahorse XF Analyzer, which measures oxygen consumption rate (OCR). The assay protocol included sequential injections of oligomycin (ATP synthase inhibitor), FCCP (mitochondrial uncoupler), and rotenone/antimycin A (electron transport chain inhibitors) to evaluate key parameters of mitochondrial function including basal respiration, ATP production, maximal respiratory capacity, and spare respiratory capacity. Data acquisition and analysis were performed using Wave software (Agilent) to generate metabolic profiles of the RPE cells under various conditions.

### 4.23 Quantitative PCR (qPCR)

qRT‐PCR was performed as previously described (Gupta et al. 2022) using the following Taqman probes (Thermo Fisher Scientific, USA): *Dct* (Mm01225584_m1), *Foxd3* (Mm02384867_s1), *Mlana* (Mm01195780_m1), *Oca2* (Mm00498969_m1), *Pax3* (Mm00435491_m1), *Rab3a* (Mm01349181_m1), *Rab37* (Mm00445351_m1), *Tyr* (Mm00495817_m1), *Atg3* (Mm00471287_m1), *Atg9b* (Mm01157883_g1), and *Uvrag* (Mm00724370_m1).

### 4.24 Statistical Analysis

All analyses were performed using the GraphPad 9.0 software. To measure significance between the experimental groups, Students' *t*‐test or ANOVA followed by Sidak's post hoc test (for experiments involving more than two groups) was performed. The significance level was set at *p* < 0.05 and all *p*‐values less than 0.05 were considered significant. The analyses were performed on at least three biological replicates (*n*) and triplicate technical replicates. Results are presented as mean ± standard deviation (SD) (Ghosh et al. 2018, 2019; Gupta et al. 2022).

## Author Contributions

Study design: D.S., S.G. Experimentation: O.C., S.B., S.G., H.L., P.S., E.M., P.G., M.N., V.K., N.S., A.S. Bioinformatic analysis: Y.X., J.Q. TEM imaging and quantification of mitochondria – melanosome propensity: J.F., M.S., K.M.K. Mass spectrometry analysis: L.R.D., R.N.C., J.W.S. Data analysis: O.C., S.B., S.G., V.S.B., P.S., H.L., K.L. Writing – original draft: O.C., S.B., S.H., J.S.Z., J.T.H., S.G., D.S. Writing – review and editing: S.C., J. – A.S., P.G.

## Conflicts of Interest

The authors declare no conflicts of interest.

## Supporting information


Appendix S1.

Figures S1–S12.

## Data Availability

All the data which were generated or analyzed during this study are included in the article and its Appendix S1. The raw files for scRNAseq and RNAseq analysis will be uploaded to the GEO NCBI database after the acceptance of the manuscript. The mass spectrometry data will be uploaded to Proteome exchange. The original uncropped western blot images and raw data for Figures 1, 2, 3, 4, 5, 6, Figures S1–S12 and Table 1 are provided in the Source Data file.
