# Supplementary material for: Activated mTOR Signaling in the RPE Drives EMT, Autophagy, and Metabolic Disruption, Resulting in AMD‐Like Pathology in Mice
Source: Aging Cell. 2025 Feb 17;24(6):e70018. doi: 10.1111/acel.70018 (PMC12151893; doi:10.1111/acel.70018)
Supplement: Supplementary file 2 — Figures S1–S12. [file ACEL-24-e70018-s001.docx]

**
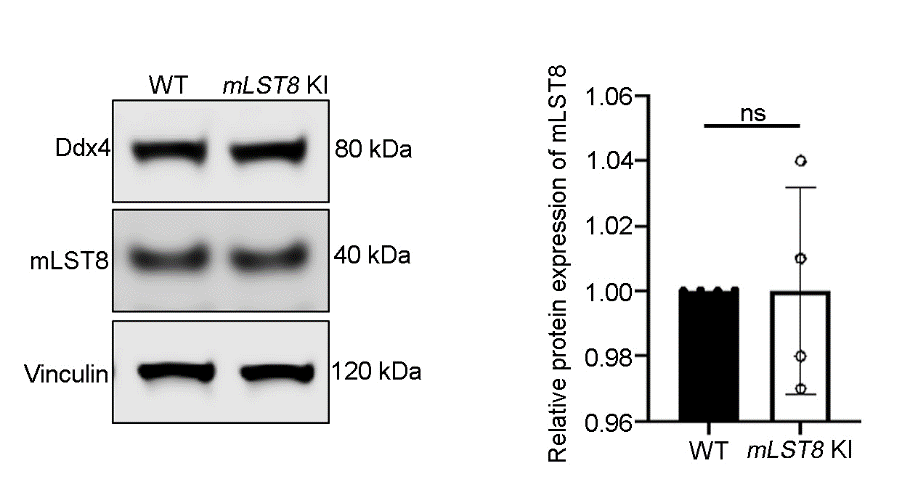
**

**Figure S1**: **mLST8 expression in testis**. Western blot showing no significant upregulation of mLST8 in the testis (organ which expresses *Best1* gene other than the RPE) of *mLST8* KI mice, compared to WT. The testis lysate was confirmed by Ddx4 (DEAD-Box Helicase 4), an ATP-dependent RNA helicase found in the chromatoid body of the germ cells and is necessary for postnatal testis development. *n*=4. ns= non-significant.


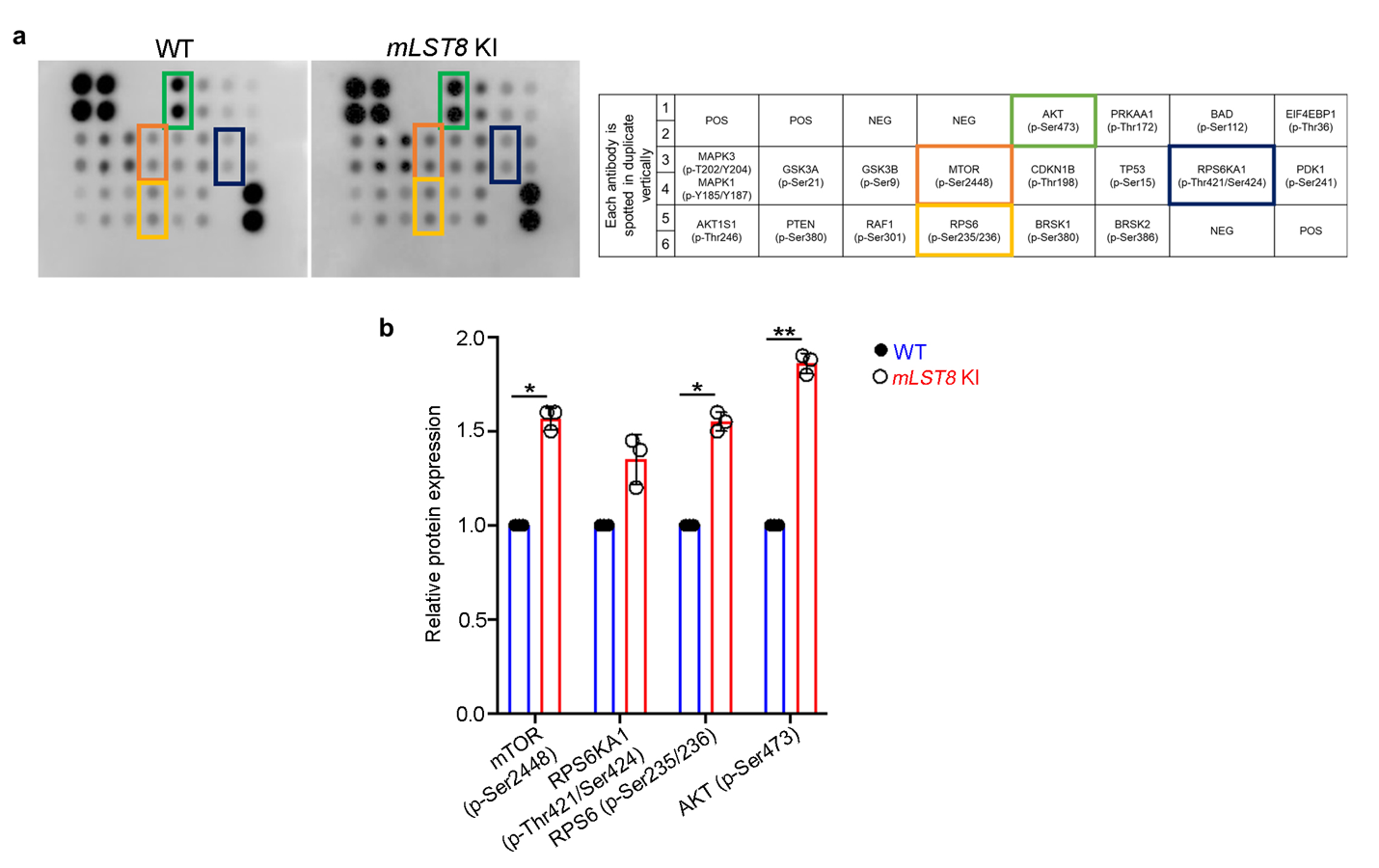


**Figure S2**: **Activation of mTORC1 and 2 target kinases in *mLST8* KI RPE cells**. (**a**) Phospho array (western blot) and (**b**) densitometry revealed significant upregulation of p-mTOR^S2448^ (orange inset), p-S6K^T421/S424^ (blue inset), p-S6^S235/236^ (yellow inset) and p-Akt^S473^ (green inset) in *mLST8* KI RPE cells, relative to controls. *n*=3. **P*<0.05, ***P*<0.01.


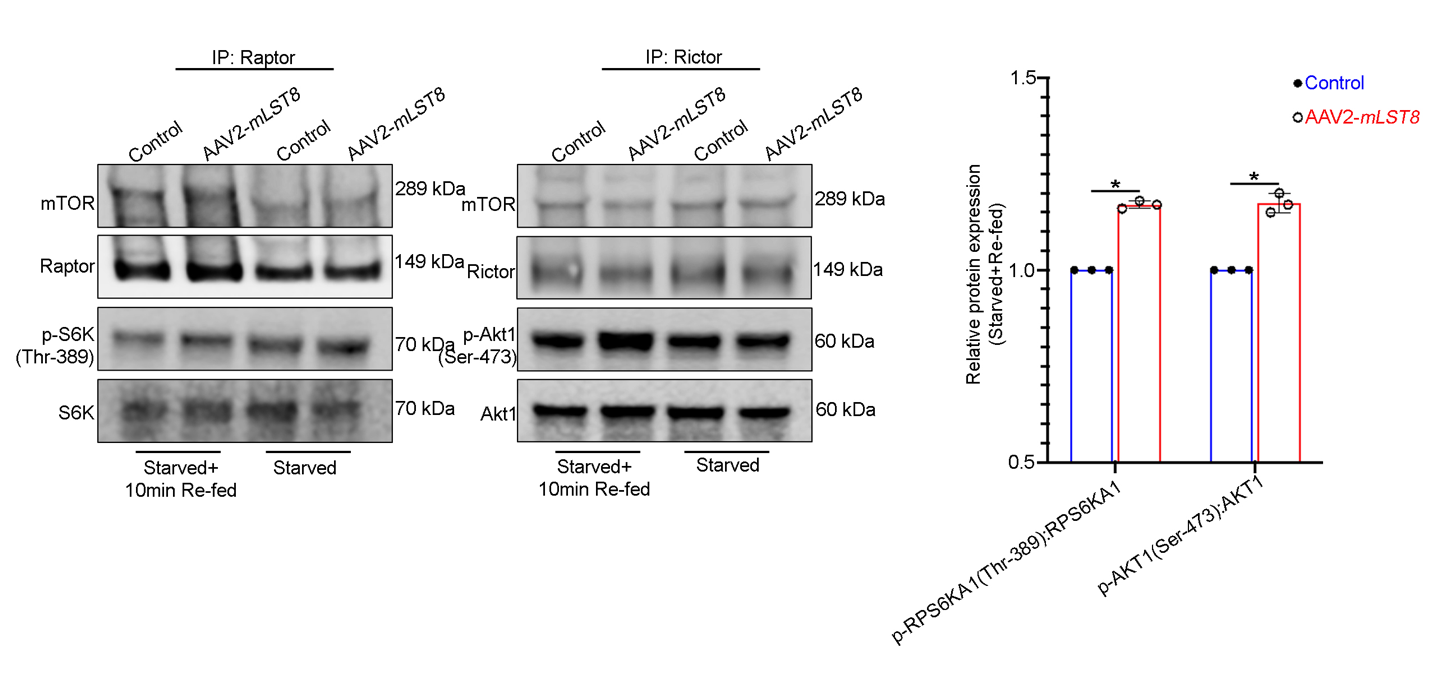


**Figure S3**: **Increased mTORC1 and 2 activities in mLST8 overexpressing ARPE19 cells**. Raptor (mTORC1 protein) or Rictor (mTORC2 protein) immunoprecipitates were prepared from control or mLST8 overexpressing (infected with AAV2-hmLST8 construct) ARPE19 cells, either starved with serum free medium overnight or starved and then re-fed with complete medium for 10 mins. The ability of Rictor and Raptor pulled complexes to phosphorylate recombinant and inactive Akt at S473 (mTORC2 target) and S6K1 at T389 (mTORC1 target), respectively were measured. Immunoblots for Rictor, Raptor and mTOR from each immunoprecipitate are shown, where the respective pulldowns showed increase in the p-Akt1/Akt1 and p-S6K1/S6K1 ratio, respectively. *n*=3. **P*<0.05.


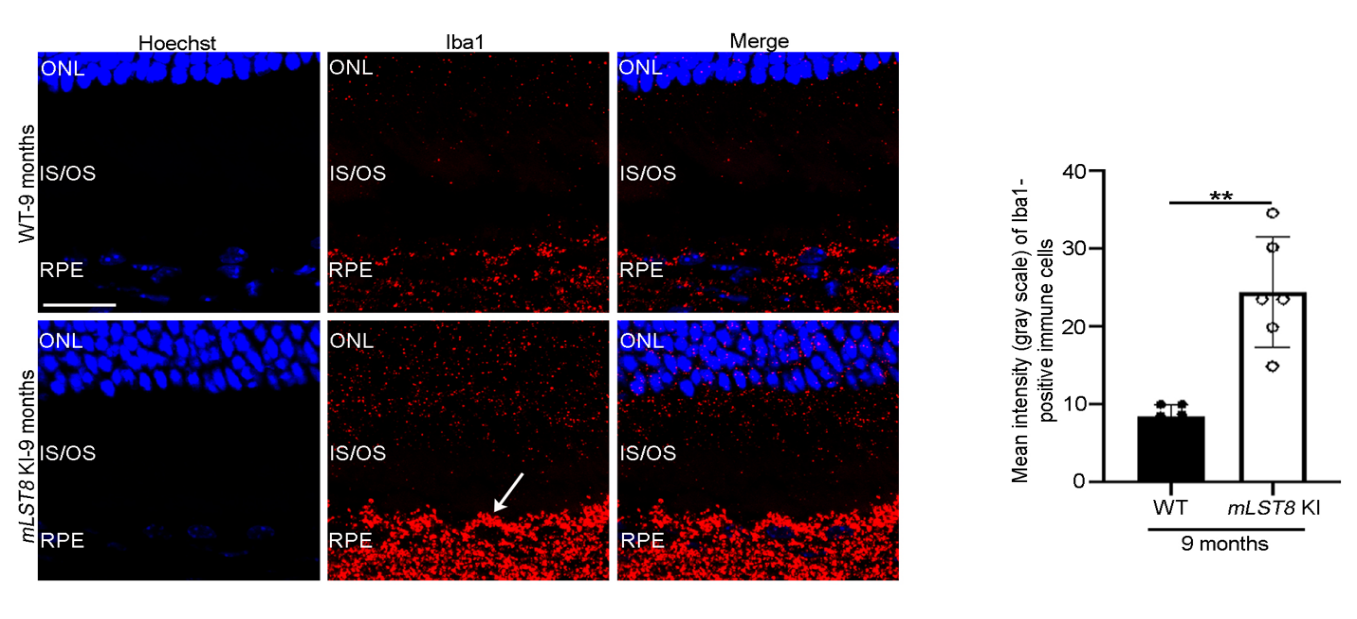


**Figure S4**: **Iba1-positive cells in the subretinal region of *mLST8* KI retina**. 9 month old *mLST8* KI retinal sections and graph denoting quantitative analysis of mean intensity shows increased Iba1-positve cells (red) shown through white arrow, compared to WT. *n*=6. ***P*<0.01. Scale bar= 10 μm.


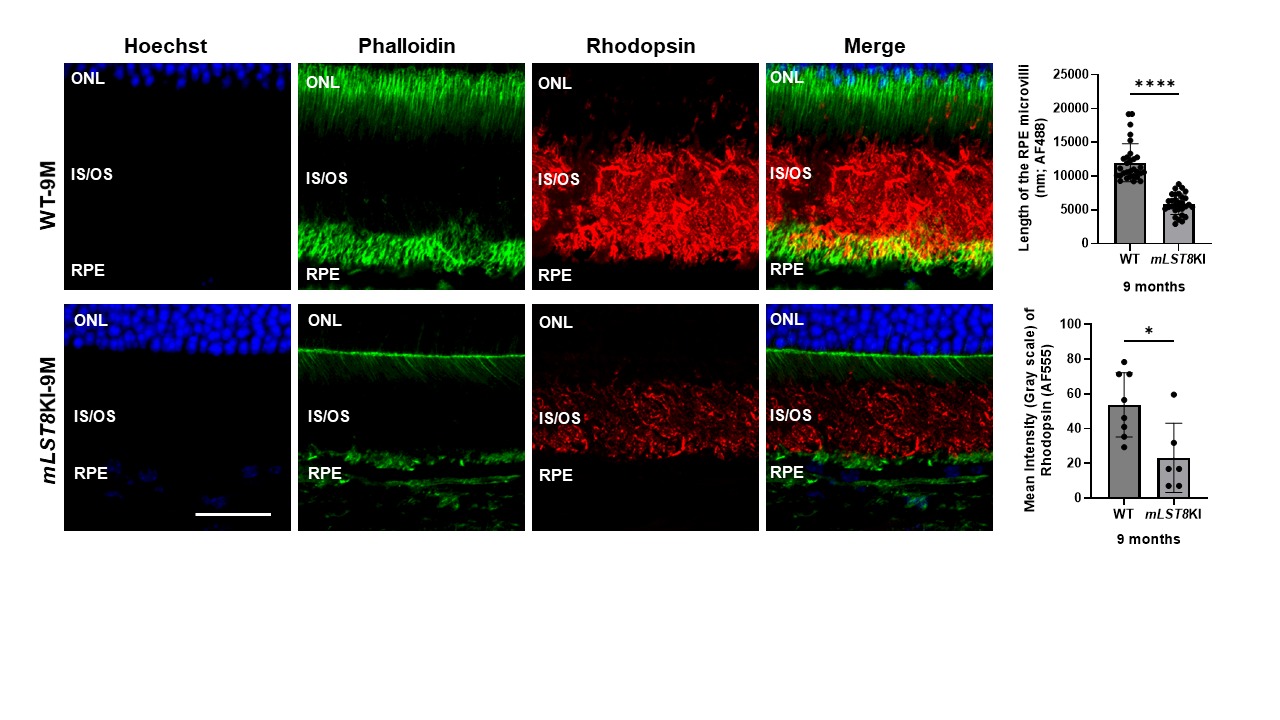


**Figure S5**: **Degenerative changes in RPE and photoreceptor in *mLST8* KI retina**. Immunofluorescence studies revealed noticeable decrease of rhodopsin (red) staining as well as discontinuity of the RPE apical microvilli (green; arrow) as well as decrease in its length in retinal sections from 9 month old *mLST8* KI mice, compared to WT. *n*=4. **P*<0.05, *****P*<0.0001. Scale bar= 10 μm


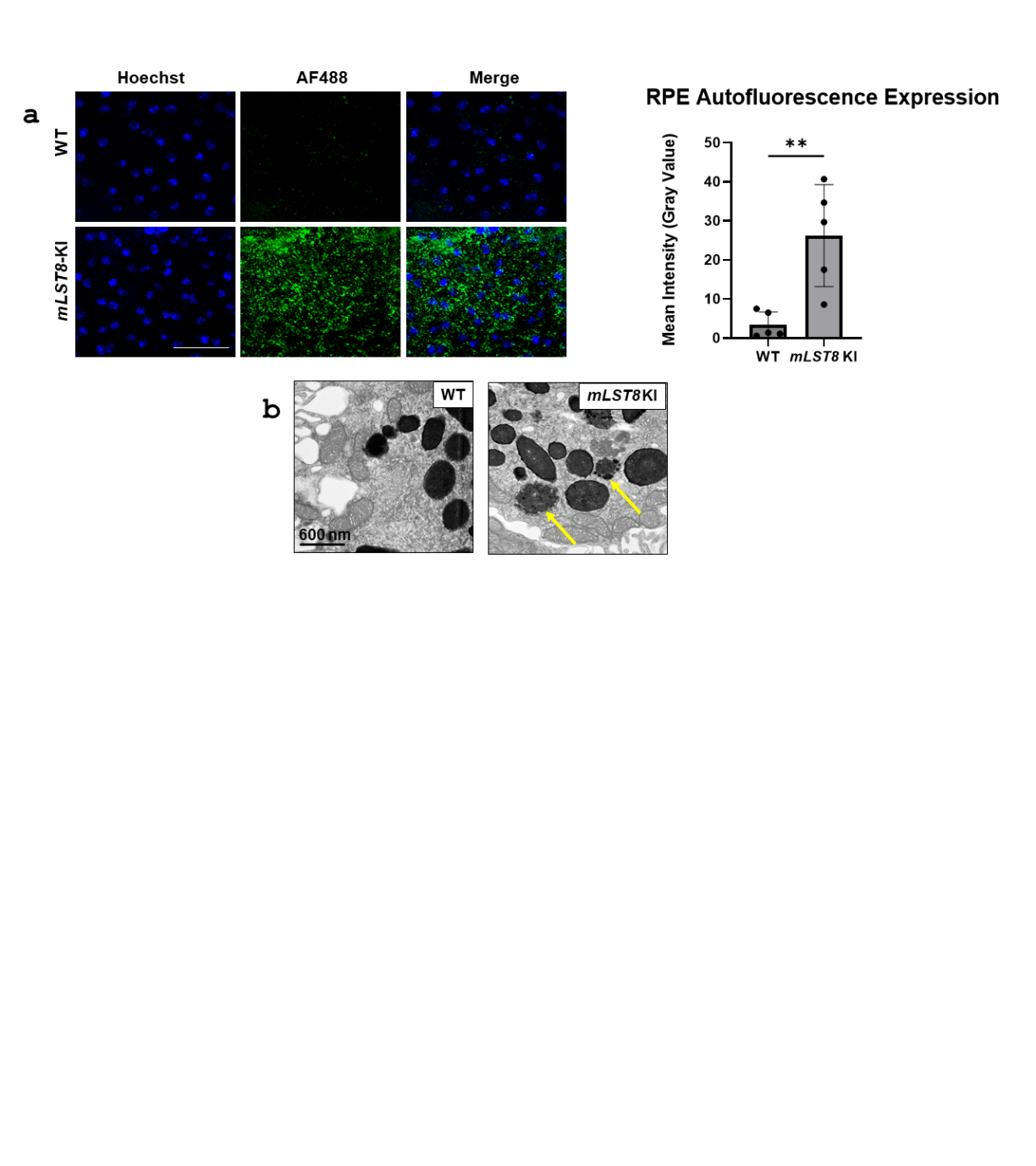


**Figure S6**: **Lipofuscin accumulation and basal laminar deposits lead to RPE dysfunction in *mLST8* KI mice.** (**a**) RPE flatmounts and quantitative analysis showing increased autofluorescence in 9 month old *mLST8* KI RPE cells, compared to age-matched WT. n=5. ***P*<0.01. Scale bar= 10 μm. **(b)** TEM images showing accumulation of lipofuscin (LF) granules in 10 month old *mLST8* KI RPE cells (arrows in **b**), but not in WT. *n*=3. Scale bar= 600 nm


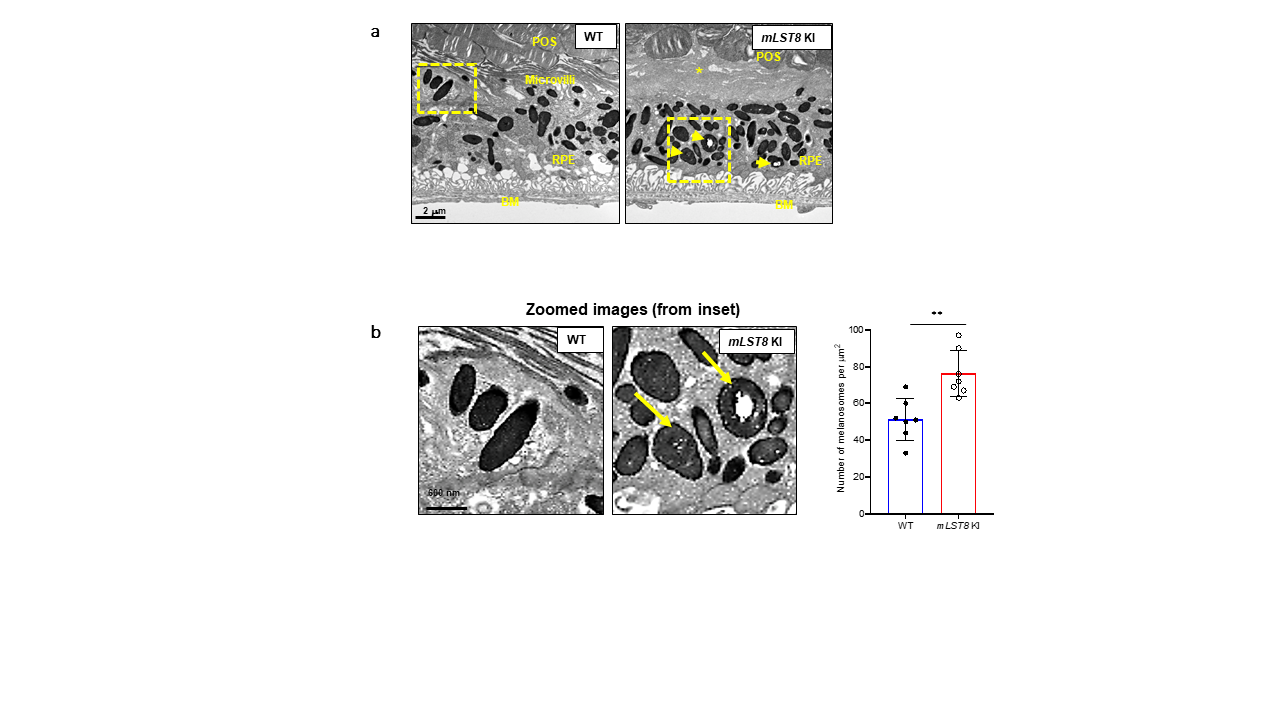


**Figure S7**: ***mLST8* KI RPE cells shows abnormal melanosome distribution**. Transmission electron micrographs of RPE cells from WT and *mLST8* KI mice at 10 months of age showing loss of microvilli (a) (asterisk), patchy melanin deposits in the melanosomes (arrows in b) as well as an increase in melanosome number in KI RPE sections, compared to WT. Seven regions per mouse were analyzed for quantification. Scale bar= 2 μm (Zoomed inset= 600 nm). *n*=3. **P*<0.05, ***P*<0.01.


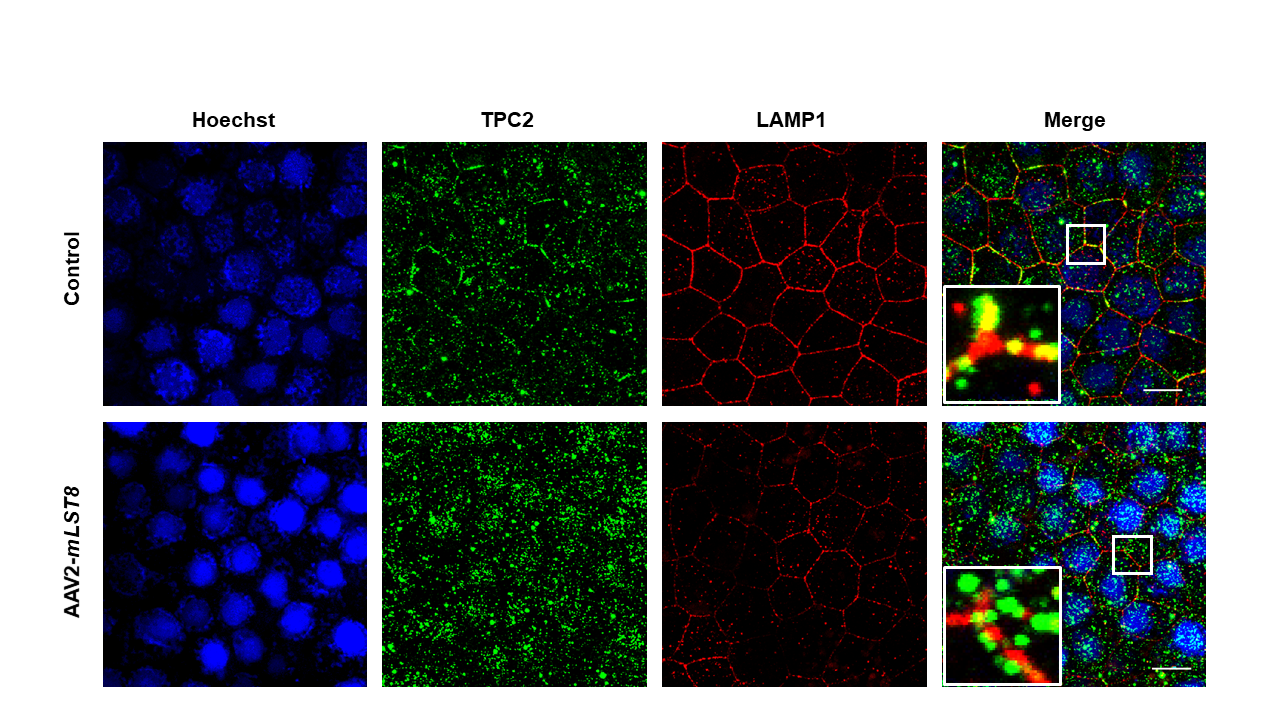


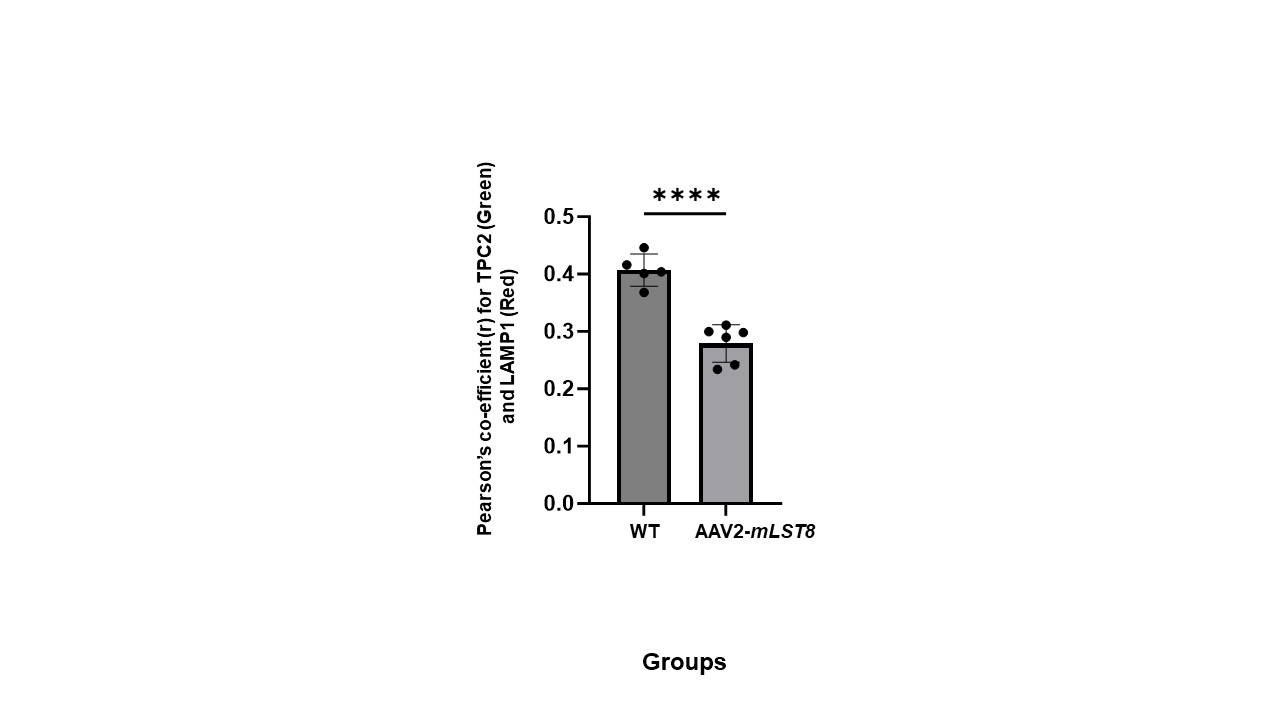


**Figure S8**: **Melanosome clearence is compromised in RPE cells overexpressing mLST8**. iPSC-derived RPE cells were infected with AAV2-mLST8 or empty vector (control) and was allowed to differentiate. This was followed by quantitative immunofluorescence assesment to evaluate the co-localization of TPC2 (matured melanosome marker) and LAMP1 (lysosome membrane protein), which showed diminished co-localization (Pearson’s co-efficient) of the two proteins in cells overexpressing mLST8. *n*=6. *****P*<0.0001. Scale bar= 10 μm (Zoomed Inset: 2 μm).


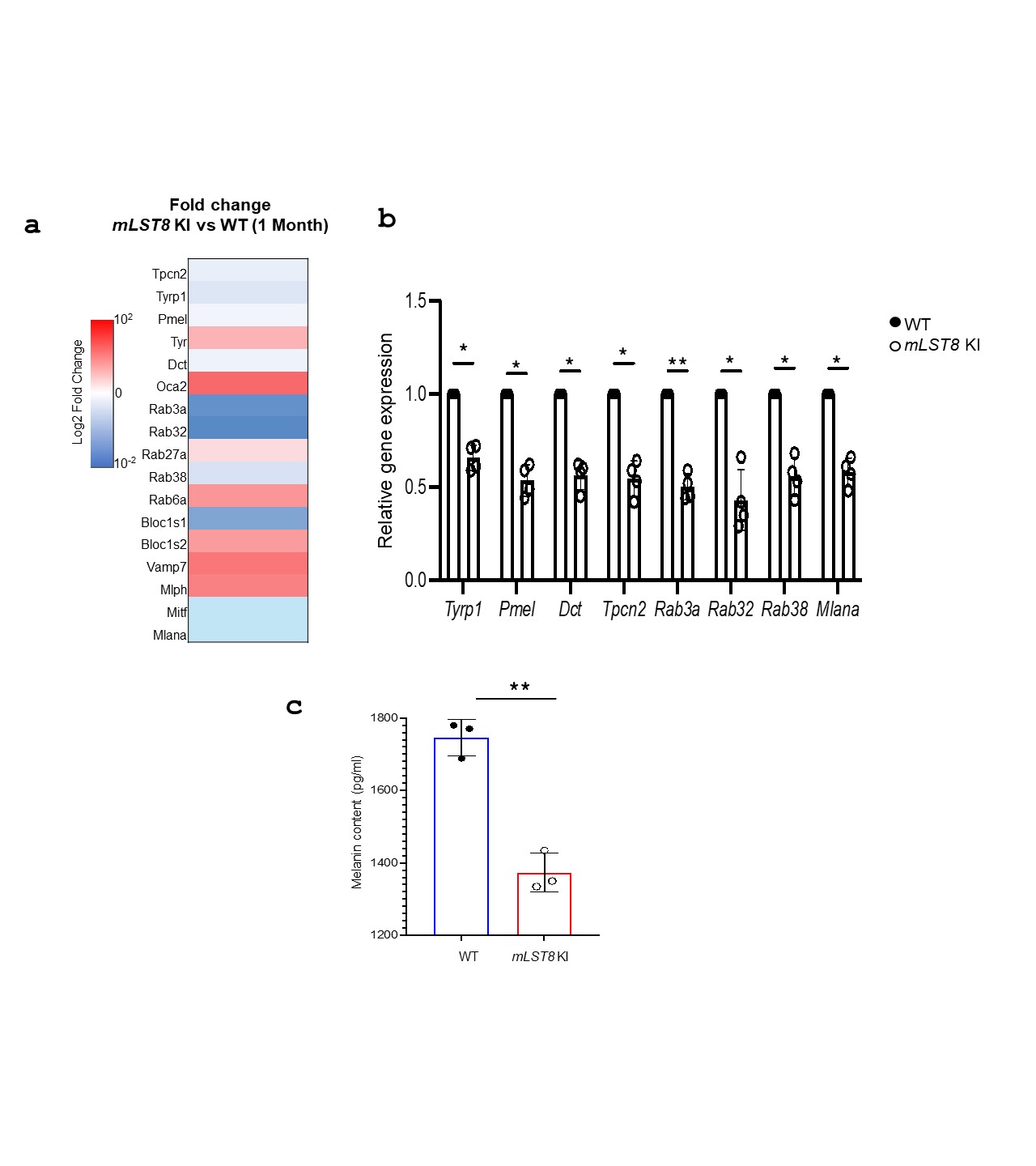


**Figure S9**: ***mLST8* KI RPE cells shows abnormal melanosome function** (**a**) RNAseq analysis from 1 month old WT and *mLST8* KI RPE cells revealed differential expression of major melanosome mediator genes. Heat map showing Log2 fold change (*mLST8* KI vs WT) for genes involved formation of melanosome function, movement and development. *n*=3. (**b**) qPCR showing significant decrease in major melanosome mediator genes like *Tyrp1, Pmel, Dct, Tpcn2, Mlana* among others in *mLST8* KI RPE cells, relative to WT. *n*=4. **P*<0.05, ***P*<0.01. (**c**) ELISA showing reduction of the total melanin content in *mLST8* KI RPE cells, compared to WT. *n*=3. ***P*<0.01.


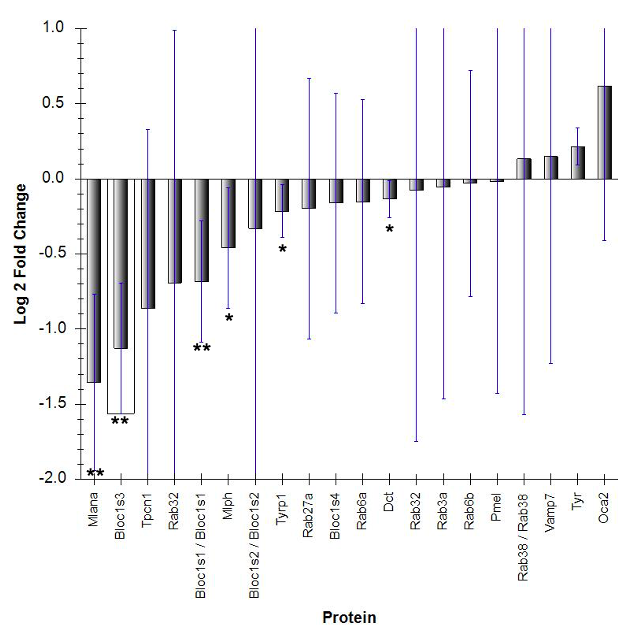


**Figure S10**: **Differential expression of melanosome-related proteins in *mLST8* KI RPE**. Graph showing fold change (*mLST8* KI vs WT; Log2 Fold change) of several melanosome related proteins in RPE lysates after quantification of DIA-based MS/MS spectra. *n*=3. ***P*<0.01, **P*<0.05.


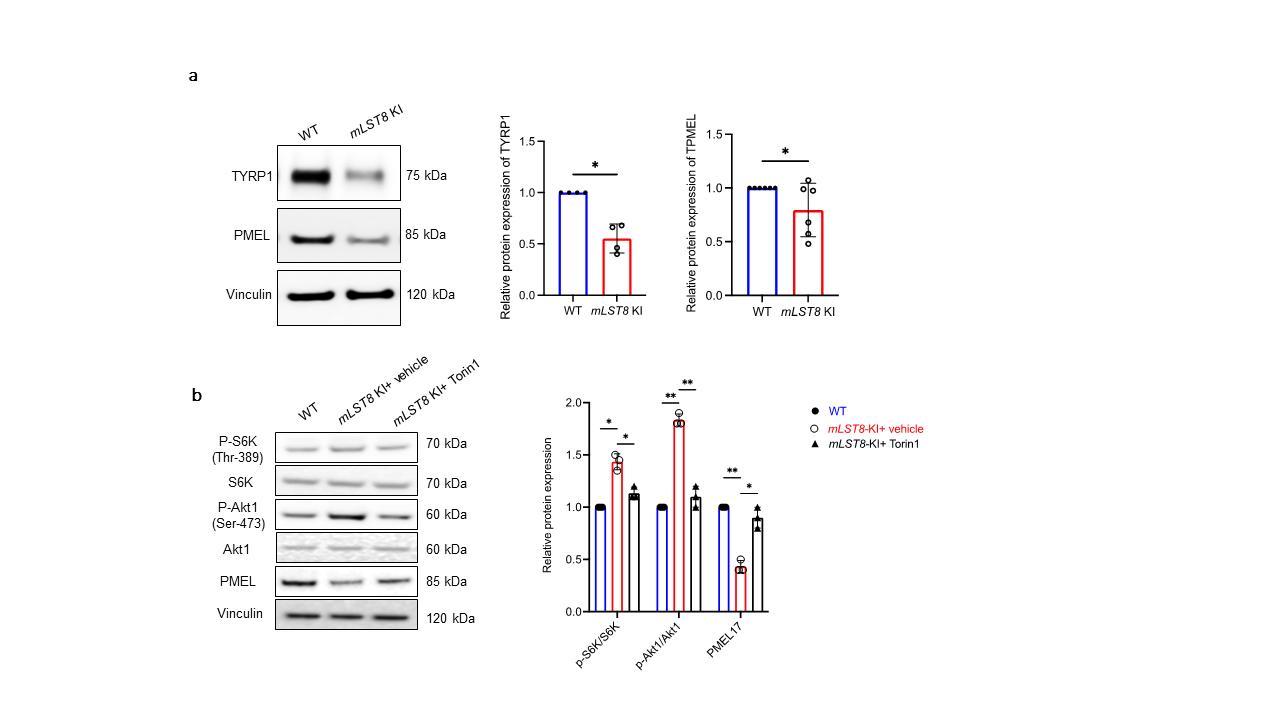


**Figure S11**: **Abnormal melanosome function in *mLST8* KI RPE is restored by treatment with Torin1.** (**a**) Western blot analysis showing decline in PMEL (structural component of melanosomes, required for its maturation) and TYRP1 (enzyme for melanin synthesis) protein levels in *mLST8* KI RPE cells, compared to WT. *n*=4 (TYRP1) and *n*=6 (PMEL). **P*<0.05. (**b**) Western blot analysis showing that Torin1 treatment (50 nM for 20 h) on *mLST8* KI RPE explants rescued the levels of the melanosome marker PMEL, as well as mTORC1 and mTORC2 targets p-S6K1^T389^ and p-Akt1^S473^. *n*=3. **P*<0.05, ***P*<0.01.


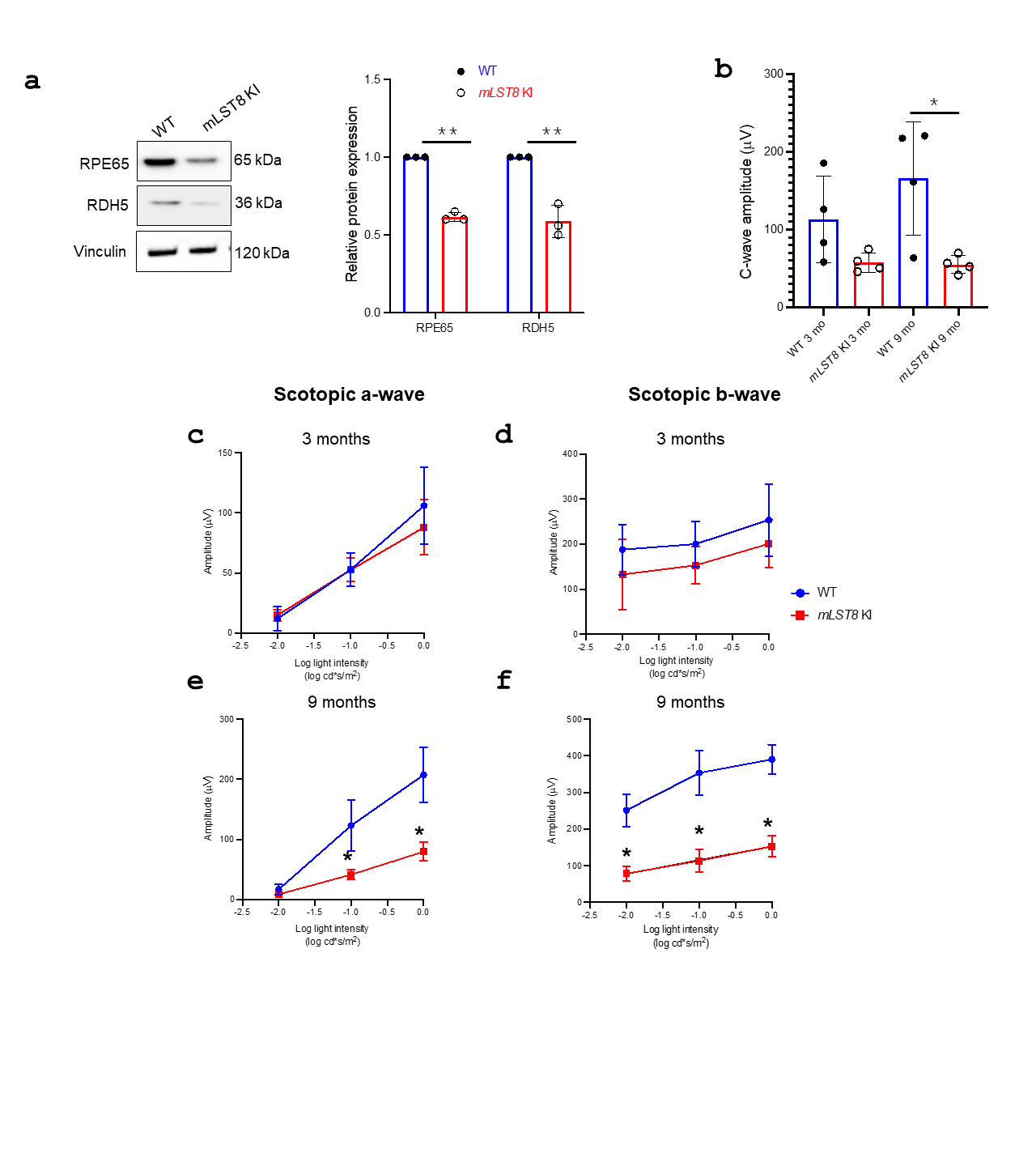


**Figure S12**: ***mLST8* KI RPE shows alterations in RPE and retinal function**. (**a**) Western blot analysis showing a decrease in visual cycle proteins RPE65 and RDH5 in *mLST8* KI RPE cells, compared to WT. *n*=3. ***P*<0.01. (**b-f**) Electroretinogram analysis showing decreased retinal function as evident from the decline in the amplitudes of c-wave (**b**), scotopic a-wave (**e**) and b-wave (**f**) in 9 month old *mLST8* KI mice, relative to WT (**b, e, f**). Such changes were not significantly seen in younger (3 months old) animals (**b, c, d**). *n*=4. **P*<0.05.
